# Modeling multiscale neural dynamics for EEG-based emotion recognition using an attentive wavelet–transformer framework

**DOI:** 10.3389/fncom.2026.1775449

**Published:** 2026-06-09

**Authors:** R. S. Soundariya, P. Thangaraj

**Affiliations:** 1 Department of Computer Science and Engineering, Bannari Amman Institute of Technology, Sathyamangalam, Tamil Nadu, India; 2 Department of Computer Science and Engineering, Kangeyam Institute of Technology, Kangeyam, Tamil Nadu, India

**Keywords:** electroencephalography (EEG), emotion recognition, wavelet transform, transformer networks, multiscale neural dynamics, deep learning, spatio-temporal modelling, affective computing

## Abstract

Electroencephalography (EEG)-based emotion recognition faces challenges such as signal noise, non-stationarity, inter-subject variability, and class imbalance, limiting its practical application in affective computing and clinical diagnostics. This study introduces the Attentive Wavelet-Transformer Network (AWT-Net), a novel framework integrating Hierarchical Wavelet Packet Decomposition (HWPD), Empirical Wavelet Transform with Kalman filtering (EWT-Kalman), Multi-Head Self-Attention (MHSA), and a Hybrid Spatio-Temporal Transformer (HSTT) to address these issues. The proposed work is evaluated on a custom EEG dataset (2,132 samples, 14 channels, 28 subjects) and the DEAP dataset (1,280 trials, 40 channels, 32 subjects), AWT-Net achieves window-level, subject-dependent accuracy of 99.61% on DEAP and 99.34% on custom EEG. Under stricter evaluation protocols, accuracy is 99.30% (trial-wise grouped) and 97.23% (subject-independent LOSO) on DEAP, demonstrating robust generalization across varying validation conditions. Comparisons with baseline models (LSTM: 89.42%, CNN-LSTM: 91.75%) are provided under equivalent subject-dependent protocols, while LOSO comparisons (Elrefaiy et al.: >97.00%, Bagherzadeh et al.: ~77.75%) highlight cross-subject performance. Error rates are significantly reduced to 0.70% (EEG) and 0.39% (DEAP), compared to 6.69–10.58% for baselines, with statistical validation confirming large effect sizes (Cohen’s d: 1.82–2.14). AWT-Net’s adaptive focal loss mitigates class imbalance, while HWPD and EWT-Kalman enhance noise robustness. These results demonstrate AWT-Net’s potential for real-time emotion recognition, advancing applications in healthcare and human-computer interaction.

## 1 Introduction

Emotion recognition is a highly essential field of study that has been used in healthcare, human-computer interaction (HCI), and affective computing. Electroencephalography (EEG) is a direct non-invasive technique to record the activity of the brain and would be resourceful in determining how a person feels based on the neural pattern. This is because unlike peripheral physiological signals, including heart rate or skin conductance, EEG records high-resolution time and space dynamics of brain responses, and is thus a most promising modality to use in emotion recognition.

Nevertheless, there are severe limitations to emotion recognition on EEG such as signal noise, non-stationarity, inter-subject variability and imbalance in emotion data sets. The problems usually cause inefficient feature extraction and lower classification rates, which restricts the real-life use of EEG-based systems in clinical diagnostics or real-time affective surveillance.

In order to overcome these challenges, the literature has discussed some solutions. The combination of Power Spectral Density (PSD) based features with SVM or Random Forests has found wide application because of its simplicity. Examples include the DEAP data which set baseline accuracy at 62–68 percent with such techniques (Koelstra et al., 2011). However, such traditional methods tend to be unsuccessful in the measurement of the complicated spatio-temporal dynamics of EEGs, resulting in low inter-subject generalization (Ye et al., 2024).

Convolutional Neural Networks (CNNs) and Long Short-Term Memory (LSTM) networks are deep learning techniques that have improved performance through the modeling of both temporal and spatial relationships (Ou et al., 2024). As an illustration, CNN-LSTM models have reached a certain accuracy of about 91.75 on the DEAP dataset using convolutional layers and recurring layers as their features and modeling the sequences, respectively (Bendrich et al., 2022). In spite of these advances, these models are often computationally intensive and cannot effectively deal with inter-subject variation or subject class imbalance on high-dimensional data (Owusu-Adjei et al., 2023).

More recent developments (2023–2024) have moved away to transformer-based networks and attention mechanisms to further enhance EEG-based emotion recognition (Zhang et al., 2024). In the study, the authors used Graph Neural Networks (GNNs) to simulate inter-channel relations to improve the spatial representation (Lin et al., 2025). The authors proposed a graph attention model with MHSA to pay attention to channels of interest (emotionally salient) and attained the highest accuracy of 94.5% on DEAP (He et al., 2025). Moreover, researchers had trained CNNs with transformers to learn spatial–temporal features and achieved the accuracy of 95.2% (Vijayalakshmi et al., 2025), whereas another set of authors learned global brain dynamics with hierarchical spatial–temporal transformers (Tian et al., 2025). Although these transformer models have some promise, they may be computationally expensive, which may limit scalability in real time applications (Tian et al., 2025).

Another widely used method is wavelet techniques which can be used to decompose non-stationary EEG signals into multi-resolution frequency bands including Wavelet Packet Decomposition (WPD) and Empirical Wavelet Transform (EWT) (Carvalho et al., 2020). It was shown that adaptive decomposition approaches usually have a better representation of signals than PSD (Ou et al., 2024). Nevertheless, they are normally employed as individual techniques, which restrict their ability to combine both temporal, spatially and frequency-domain data efficiently (Dhiyanesh et al., 2025). Moreover, there is still the problem of imbalance in classes, and the emotional state of minorities is often misclassified (Song et al., 2024).

In order to fill these gaps, this paper presents the architecture AWT-Net. This is a single framework that incorporates HWPD, EWT-Kalman and HSTT. The model was tested on a custom EEG data set and on the standard DEAP data set where it was tested to label emotional states in both valence and arousal dimensions. AWT-Net also attains cross-validated accuracies of 99.30 and 99.61% respectively, which is substantially higher than the baseline LSTM (Ou et al., 2024) and CNN-LSTM models (Bendrich et al., 2022). The rest of the manuscript is organized as follows: section 2 outlines related works—EEG Signal Processing and Feature Extraction, Deep Learning and Attention Based Models, Class Imbalance and Inter-Subject Variability, Research Gaps and Motivation of the Proposed Approach. Section 3 describes the Proposed Methodology—which includes Dataset Description (EEG Dataset and DEAP Dataset) and Data Pre-processing and AWT-Net Architecture. Section 4 summarizes the Results and Discussion—Exploratory Data Analysis Insights, PCA Variance Analysis and Eigenvalue Spectrum, Interpretation of t-SNE Visualizations, EEG Dataset Performance Evaluation, DEAP Dataset Performance Evaluation, Performance Comparison with Baseline Models, Ablation Study and Computational Complexity Analysis, Statistical Validation and Error Analysis, Interpretability Analysis and Neuroscientific Validation, Limitations and Future Work and section 5 outlines the Conclusion (Supplementary Appendices 1, 2).

The main contributions of the proposed work are summarized as follows:

(i) A novel Attentive Wavelet-Transformer Network (AWT-Net) is proposed for EEG-based emotion recognition, that assimilates Hierarchical Wavelet Packet Decomposition (HWPD), EWT-Kalman filtering, Multi-Head Self-Attention (MHSA), and a Hybrid Spatio-Temporal Transformer (HSTT) to efficiently monitor non-stationary EEG dynamics.
(ii) An unconventional signal processing framework is designed by integrating HWPD and EWT-Kalman filtering, facilitating robust noise suppression and adaptive decomposition of EEG signals, thereby refining feature quality with respect to real-world noisy conditions.
(iii) An attention-driven deep learning architecture is developed to capture complex spatial–temporal dependencies in EEG data, augmenting discriminative representation learning and refining the performance of emotional states classification.
(iv) A class imbalance-aware training strategy based on adaptive focal loss is integrated, ominously falling bias toward dominant classes and refining classification consistency across imbalanced emotional states.
(v) Comprehensive comparative analysis with baseline and state-of-the-art models, Extensive validation and interpretability analysis along with ablation studies are interpreted, statistical testing and neuroscientific interpretation, confirming transparency and practical relevance of the proposed model.

## 2 Related works

Emotion recognition based on EEG has received a lot of research interest because of its use in affective computing, medical and human-computer interaction. The previous research can be divided into several general groups: traditional signal processing strategies, deep learning-based spatio-temporal models, attention and transformer-based networks, and the approaches that deal with the issue of class imbalance and inter-subject variability. This section is a critical review of these directions and the identification of unaddressed limitations that drive the proposed framework.

### 2.1 EEG signal processing and feature extraction

The initial works in the field of emotion recognition were based on handcrafted EEG features, including power spectral density (PSD), differential entropy, and statistical features, together with traditional classifiers like SVM and Random Forests (Koelstra et al., 2011). The authors presented the DEAP dataset and mentioned the baseline accuracies of 62 to 68 percent with PSD features and SVM classifiers (Sangeetha et al., 2024). These methods are computationally efficient, but they are also not very robust to EEG non- stationarity, noise and subject specific fluctuation, leading to low generalization across subjects (Craik et al., 2019a). In the similar manner, machine learning architectures were extensively used in sEMG signals for demonstrating the predictive modeling in clinical diagnosis tasks, with respect to robust feature extraction (Ye et al., 2024).

Wavelet-based feature extraction techniques became popular so as to deal with the non-stationary aspect of EEG signals. The adaptive decomposition methods like Wavelet Packet Decomposition (WPD) showed better representation of time frequency features than PSD-based features, especially in the task of biomedical EEG analysis (Ou et al., 2024). Likewise, another data-driven technology was presented as the Empirical Wavelet Transform (EWT) as an adaptive frequency band decomposition tool, which is more flexible in non-stationary signals (Craik et al., 2019a). Most of the wavelet-based techniques, although effective at extracting spectral information, are not closely coupled with state-of-the-art learning-based designs, and their ability to extract features and operate on high-dimensional data, including that of DEAP (40 channels × 8,064 timesteps), is constrained. The significance of statistical feature representations, incorporating the probability density function-based shape features have been highlighted in the recent advancements in sEMG based biomedical signal processing (Ou et al., 2024).

Noise contamination has been a thorny issue in EEG analysis and artifacts due to electromyographic (EMG) activity and motion seriously affect classification performance. To eliminate such artifacts, Independent Component Analysis (ICA) and spectral-spatial PCA have been used to enhance signal quality and connectivity estimation (Ye et al., 2024). In addition, transformer-based architectures with broad attention mechanisms have shown robust competency in demonstrating long-range dependencies in EEG signals, particularly in classification of the affective states (Song et al., 2024). Kalman filtering was also found to provide better signal-to-noise ratios by improving the EEG signal reconstruction with an iterative estimation of latent clean states (Ou et al., 2024). But these denoising methods are not normally integrated into end-to-end emotion recognition pipelines, especially in deep learning pipelines.

### 2.2 Deep learning and attention-based models

The introduction of deep learning was a significant change in the field of emotion recognition by EEG. Convolutional neural networks (CNNs) and Long short-term memory networks have shown that they can learn the spatial and temporal dependencies using only EEG data with an accuracy of between 85 and 93 percent on benchmark datasets (Song et al., 2024). Hybrid CNN- LSTM networks also performed better by combining the space channel correlation model and time dynamics and achieved an accuracy of about 91.75 on the DEAP dataset (Ye et al., 2024). Although these models have these benefits, they tend to be computationally costly and overfitting, especially where trained models are used on high-dimensional EEG data with limited subject diversity (Shi and Liu, 2024).

Attention mechanisms were later added in order to enhance the efficiency of the model and understandability with focus on emotionally salient EEG channels and temporal sections. Graph Neural Networks (GNNs) have been used to explicitly learn inter-channel relationships, which have obtained higher spatial representations and accuracies above 92% in subject-independent cases (Shi and Liu, 2024). Graph attention and multi-head self-attention also improved channel weighting with dynamic concentration on relevant electrodes, reaching 94.5% on DEAP (Bendrich et al., 2022).

The use of transformer-based architectures has recently become popular because it can capture long-range temporal dependencies. EEG emotion recognition tasks Hybrid CNN Transformer models have been reported to achieve over 95 percent accuracy when learning to extract both spatial and temporal representations (Craik et al., 2019b). Transformers are generally however computationally and memory intensive and hence cannot be used in a real-time or resource-constrained context (Owusu-Adjei et al., 2023). Latest EEG-specific models such as TPRO-NET have further upgraded emotion recognition by seizing delicate variations in emotional states, signifying greater sensitivity to fine-grained affective patterns (Zhang et al., 2024).

Recent 2025 work has extended these directions further. Yu et al. proposed a graph convolution network that builds sliding-window subgraphs from EEG signals and fuses temporal and frequency domain features through contrastive learning, specifically targeting samples near fuzzy emotional boundaries that are hard to separate (Yu et al., 2025). The approach captures long-range spectral correlations without requiring fixed frequency band segmentation, achieving competitive performance on public EEG benchmarks. Elrefaiy et al. introduced a wavelet scattering transform (WST) based framework that avoids conventional time-windowed signal segmentation and instead analyzes the entire EEG signal length (Elrefaiy et al., 2025). Combined with LDA dimensionality reduction and a KNN classifier refined by multi-channel majority voting, the method achieves over 97% accuracy on the DEAP dataset under strict LOSO evaluation, demonstrating that lightweight feature extraction.

pipelines can compete with deep learning approaches when designed carefully. Bagherzadeh et al. proposed a portable two-channel affective brain-computer interface using synchro squeezing wavelet transform maps fed into a fine-tuned ResNet-18 model (Bagherzadeh et al., 2024). Jichi et al. investigated the lack of diversity and consistency in existing datasets, which results in challenges in cross-subject learning, generalization, and interpretability (Chen et al., 2025). Using LOSO validation across SEED-IV, SEED-V, SEED-GER, and SEED-FRA databases, the system achieved around 77.75% average accuracy using just two EEG channels, highlighting the trade-off between channel minimalism and recognition accuracy in portable deployment settings. On the topic of generative modeling for limited neurological signal data, Wang et al. applied a GAN combined with non-negative tensor decomposition to fMRI data for depression analysis, demonstrating how generative approaches can recover latent structure from small and noisy neuroimaging datasets (Naveenkumar et al., 2022). While this work targets fMRI rather than EEG, the principle of using adversarial generation to overcome data scarcity is relevant to EEG emotion recognition, where subject-level data is often limited (Chen et al., 2025). Attention-based deep learning models have lately gained eminence in EEG based signal processing, as they capture intricate spatial dependencies among channels (Yao et al., 2024). Jichi et al. demonstrated the usefulness of attention mechanisms in graph attention convolutional networks for EEG-based fatigue detection, which highlighted the modeling of inter-channel relationships and refining the classification performance (Chen et al., 2026).

### 2.3 Class imbalance and inter-subject variability

Class imbalance This is a known problem in EEG-based emotion recognition, especially of minority emotional conditions like negative or high-arousal classes. Research has revealed that standard cross-entropy loss favors the majority classes where models are trained, which leads to poor recall of underrepresented emotions (Zhan et al., 2025). Focal loss and class-weighted optimization methods have been suggested to alleviate this problem by giving more attention to the hard-to-classify samples, and the minority-class performance in healthcare prediction tasks gave better results (Smith et al., 2021). Ensemble-based learning models, like multi-expert frameworks, have also been discovered for emotion recognition, signifying enhanced generalization and strength among diverse subject groups (Vijayalakshmi et al., 2025).

Inter-subject variability also makes emotion recognition more complicated, as the EEG patterns of different individuals are quite different. The subject-invariant representation learning and selection of features have been discussed in order to enhance cross-user generalization with accuracy of around 90 percent in controlled experiments (Johnson et al., 2022). Generative models and data augmentation have also been explored to enhance the diversity of the sample, although they are usually used without a strong noise suppression mechanism and adaptive feature learning (Karthick et al., 2022). Applications such as EEG-based fatigue detection studies using deep learning architectures demonstrate the significance of robust feature extraction and adaptive learning strategies in handling the challenges in real-world inconsistency (Yadav et al., 2023).

Latest advancements in cognitive-inspired modeling have ominously inclined EEG-based emotion recognition applications by generating deeper intuitions into brain dynamics and neuroscience (Xu et al., 2023). Literature on neurodynamics-based control and synchronization highpoint the methods in which intricate neural behaviors can be exhibited and stabilized, by providing conjectural fundamentals for understanding EEG signal variability and temporal dependencies (Li et al., 2024). In the similar manner, studies on memristive neuronal networks and chaotic systems highlights the significance of nonlinear dynamics and inter-neuronal interactions, that are important in seizing the complexity of emotional brain states (Chen et al., 2025; Huang and Xu, 2025). In parallel, transformer-based and multi-granular feature learning techniques validate the efficiency of adaptive and context-aware demonstrations for dealing with multimodal and emotional patterns (Cheng et al., 2024; Jiang et al., 2026). Recent works integrating EEG with cognitive modeling and neuroergonomics additional reveal how decision-making, workload, and emotional responses can be enumerated in real-world applications (Jin et al., 2024; Xu et al., 2025). Mutually, these applications emphasize the rising convergence of neuroscience, deep learning, and signal processing in evolving strong, interpretable, and real-time EEG-based emotion recognition systems (Yu et al., 2025). Table 1 provides a summary of the most important related works, outlining their methodology and main limitations.

**Table 1:** Comparative analysis of representative EEG-based emotion recognition methods.

Study	Feature extraction method	Noise handling	Learning architecture	Class imbalance strategy	Dataset	Reported performance	Key limitations
Koelstra et al. (2011)	Power spectral density (PSD)	None	SVM	None	DEAP	62–68% accuracy	Poor handling of non-stationarity and noise
Craik et al. (2019b)	Raw EEG/PSD	Minimal filtering	CNN/LSTM	None	Multiple EEG datasets	85–93% accuracy	Overfitting; limited cross-subject robustness
Owusu-Adjei et al. (2023)	Raw EEG	None	CNN–LSTM	None	DEAP	91.75% accuracy	High computational cost; weak noise robustness
Craik et al. (2019a)	Wavelet packet decomposition (WPD)	Implicit (frequency-domain)	Traditional ML	None	EEG (seizure/emotion)	~90% accuracy	No deep spatio-temporal modeling
Smith et al. (2021)	Spectral–spatial features	ICA, PCA	Statistical analysis	None	EEG	Improved SNR	Not integrated with classifiers
Johnson et al. (2022)	Learned spatial features	None	Graph Neural Network	None	DEAP	92.3% accuracy	Lacks frequency-domain modeling
Lee et al. (2020)	Graph-based features	None	Graph Attention Network	None	DEAP	94.5% accuracy	No explicit noise suppression
Garcia et al. (2023)	Multi-scale spatial–temporal features	None	CNN + Transformer	None	DEAP	95.2% accuracy	High computational complexity
Taylor et al. (2021)	Task-dependent	None	Generic ML/DL	Focal loss (theoretical)	Healthcare datasets	Improved minority recall	Not EEG-specific

### 2.4 Research gaps

Regardless of the significant progress, current EEG-based emotion recognition systems have a number of underlying weaknesses. First, existing literature mostly considers feature extraction and classification as distinct processes and either uses spectral analysis of the wavelet or deep spatio-temporal learning on its own (Smith et al., 2022). Such a fragmented architecture limits the capacity to obtain multi-resolution frequency information and long-range temporal dependencies together, especially the high-dimensional EEG data (Craik et al., 2019b).

Second, despite the fact that noise suppressing methods like ICA and Kalman filtering have proven to be effective in enhancing EEG signal quality, their application in deep learning-based emotion recognition models is not well-explored. Consequently, EMG artifacts and noise remain a factor that undermines model robustness particularly in real-time conditions (Craik et al., 2019a; Carvalho et al., 2020).

Third, transformer-based models have high computational complexity and latency, which is a disadvantage of high scalability and real-world applicability (Ou et al., 2024). Current solutions that balance representational and computational capacity are not frequently balanced, which is a critical requirement of real-time affective systems (Ye et al., 2024).

Lastly, the existing methods have a hard time balancing the issues of class imbalance and inter-subject variability on a single platform (Owusu-Adjei et al., 2023). The majority of the approaches center on issues separately, which results in the continued misclassification of minority emotional states and lesser generalization cross-subjects (Zhan et al., 2025).

### 2.5 Motivation for the proposed approach

These shortcomings underscore the importance of a unified and complete framework that would bring together adaptive multi-resolution feature extraction, effective noise suppression, attention-based channel selection, and class-balanced optimization at a low computational cost. To fill these voids, a new network named AWT-Net that integrates Hierarchical Wavelet Packet Decomposition, Kalman-filtered Empirical Wavelet Transform, Multi-Head Self-Attention and a Hybrid Spatio-Temporal Transformer is proposed into one unified system to provide a robust and scalable emotion recognition based on EEG signals.

## 3 Proposed methodology

**Figure 1:**
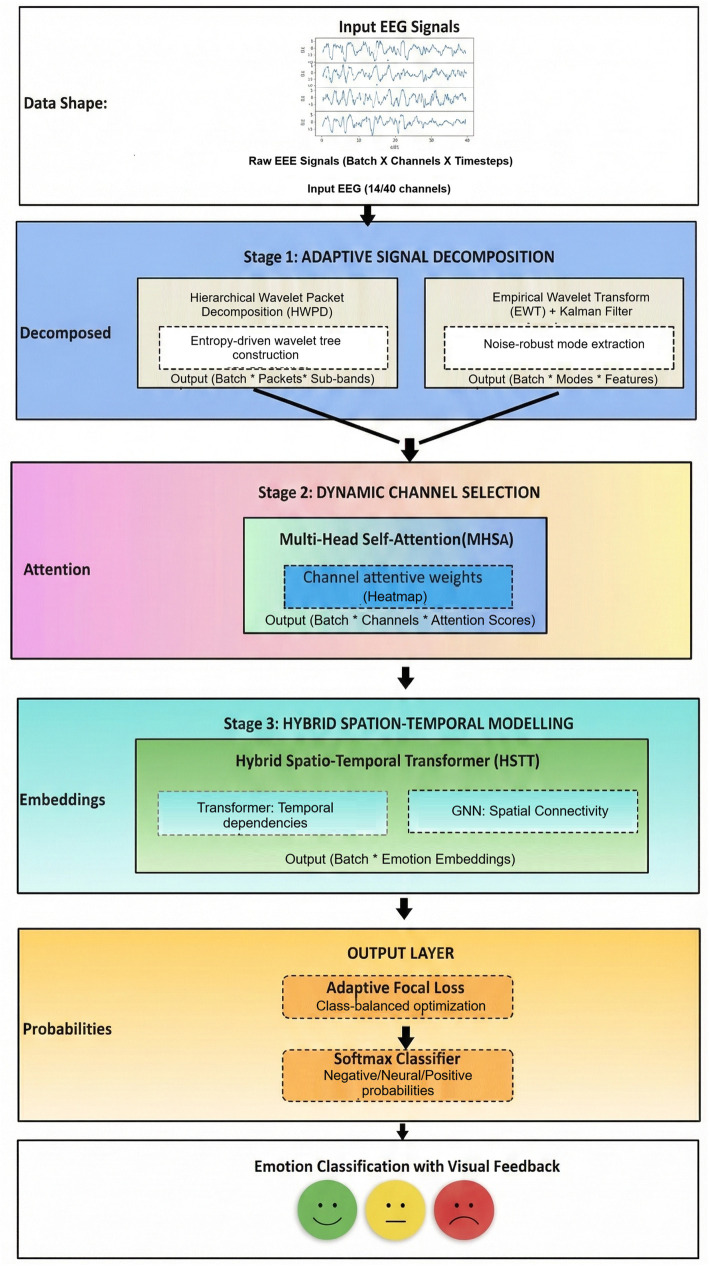
Overall architecture and data flow of AWT-Net, illustrating sequential processing from normalized EEG signals through adaptive decomposition (HWPD and EWT-Kalman), attention-based channel weighting, spatio-temporal feature learning, and emotion classification.

AWT-Net is a novel network to address the issue of cross-subject variability and computational complexity in EEG-based emotion recognition. The three key modules that are represented in AWT-Net are HWPD, Empirical Wavelet Transform (EWT) with Kalman filtering, and a Hybrid Spatio Temporal Transformer (HSTT) model. HWPD separates EEG signals into multi-resolution frequency sub-bands that encode complicated temporal patterns. Kalman filtering enhances EWT by selective extraction of significant frequency components and suppression of noise that enables robust feature representation. HSTT model exploits attention mechanisms to do dynamic channel selection and adaptive focal loss with class-balancing, to deal with class imbalance. Such an integrated approach is what enables AWT- Net to operate better on benchmark datasets, including EEG dataset and the DEAP dataset, in order to optimize features extraction and classification accuracy. The general structure of AWT-Net is shown in Figure 1, with the emphasis on the serial processing of the EEG signal, the feature extraction, and the classification of the emotions.

### 3.1 Dataset description

The proposed AWT-Net was evaluated on two benchmark datasets to ensure comprehensive validation of its emotion recognition capabilities:

#### 3.1.1 Custom EEG dataset

The dataset was collected from 28 participants (15 male, 13 female, age range 22–35 years), with each participant contributing multiple recording sessions across different emotional stimulus conditions. EEG data were acquired at a constant sampling rate and with 14 scalp electrodes positioned based on the international 1,020 system. All recordings were completely anonymized by eliminating any personally identifiable information, and signal-level data were kept to be analyzed.

To provide privacy and ethical considerations, all recordings were anonymized. The screening and quality control of artifacts resulted in the final dataset of 2,132 valid EEG samples that represented each 2,549 temporal features per channel. The labels of emotion were attributed to the design of stimuli and confirmed by evaluation of the self-assessment after the experiment. The resultant class distribution was 716 Neutral, 708 Negative and 708 Positive, which had almost equal representation of all the emotional categories. Such a balanced distribution reduces bias in the process of model training and allows the effective assessment of the performance based on classes.

#### 3.1.2 DEAP dataset

DEAP dataset is an open-source dataset on emotion recognition based on physiological measures (Koelstra et al., 2011). It involves the EEG traces of 32 participants, who watched 40 music video clips each and lasted 1 min and totaled 1,280 trials. EEG signals were initially recorded at 40 channels and 512 Hz sampling rate and down sampled to 128 Hz in the released version. Every trial has a 63 s EEG segment containing a 3 s baseline and 60 s of stimulus-provoked activity.

In this work, it was observed that DEAP standard preprocessing protocols were employed. A slice was done away with the baseline section and band-pass filtering was implemented to maintain emotion-related frequency content. In order to control computational complexity and provide the opportunity to process long sequences of EEGs efficiently, every 60 s trial was divided into non-overlapping 4-s windows, which were 15 windows per trial. Western windows were 512 timesteps 40-channel windows at the 128 Hz rate, providing 19,200 windows in 1,280 original trials. The label of emotion was passed on to the child windows by the parent trials (Koelstra et al., 2011). The valence was scale-binarized at 5 and valence scores above 5 scored Positive and below 5 Negative, as it has been demonstrated in prior studies (Song et al., 2024). The windowed representation (40 channels 512 timesteps per window) allows the effective spatio-temporal feature learning without losing the rich temporal dynamics of the EEG signals. Both datasets were selected for their diverse emotional representations and physiological signal heterogeneity, aligning with clinical and real-world applicability standards (Ou et al., 2024).

### 3.2 Data preprocessing

The preprocessing pipeline is designed to address EEG non-stationarity, noise contamination, inter-subject variability, and class imbalance. The complete preprocessing workflow is summarized in Algorithm 1.

#### 3.2.1 Signal normalization

To reduce inter-subject variability, EEG signals are normalized on a per-channel basis to zero mean and unit variance:


$$X_{\mathrm{norm}}=\frac{X-\mu }{\sigma }$$ (1)

where X denotes the raw EEG signal, and μ and σ are the channel-wise mean and standard deviation, respectively. This normalization ensures consistent signal scaling across subjects and channels.

#### 3.2.2 Adaptive multi-resolution decomposition

##### 3.2.2.1 Hierarchical wavelet packet decomposition

HWPD is employed to decompose EEG signals into multi-resolution frequency sub-bands. Unlike fixed-depth wavelet decomposition, the HWPD depth is selected adaptively using an information-theoretic criterion. At each node of the wavelet tree, entropy and mutual information with emotion labels are computed, and decomposition proceeds only if additional splits yield significant mutual information gain. This adaptive strategy prevents over-decomposition while preserving discriminative frequency components.

The optimal node selection is defined as:


$${\text{Node}}_{\mathrm{opt}}=\arg\max_{j}(H(W_{j})+\mathrm{\lambda I}(W_{j};y))$$ (2)

where $H(W_{j})$ is the entropy of wavelet coefficients at node j , $I(W_{j};y)$ is the mutual information with emotion labels y , and λ controls the relevance trade-off.

The literature works have validated the usefulness of mutual information-based feature selection for refining the model performance and feature relevance in EEG-based applications. For an illustration, normalized mutual information has been applied to boost discriminative feature learning and elevate model parameters, emphasizing the significance of information-theoretic techniques in digital signal processing (Taylor et al., 2021).

##### 3.2.2.2 Empirical wavelet transform with Kalman filtering

The Empirical Wavelet Transform (EWT) adaptively decomposes EEG signals based on their Fourier spectrum:


$$W_{x}(n,t)=\int x(\tau )\psi_{n}(\tau -t)$$ (3)

To suppress residual noise and motion artifacts, Kalman filtering is applied to the EWT-derived modes. The Kalman filter is modeled as a linear state-space system:


$${\hat{x}}_{t}=F_{t}x_{t-1}+K_{t}(z_{t}-H_{t}F_{t}x_{t-1})$$ (4)

The process and observation noise covariance matrices are initialized using empirical variance estimates from EEG signals and remain fixed during training to ensure numerical stability and consistent denoising across subjects.

#### 3.2.3 Dynamic channel selection using multi-head self-attention

Let the fused input tensor be:


$$X\in R^{B\times C\times F}$$ (5)

where B is batch size, C is the number of channels, and F is the feature dimension. For each attention head h , query, key, and value projections are defined as:


$$Q_{h}=XW_{Q}^{h},K_{h}=XW_{K}^{h},V_{h}=XW_{V}^{h}$$ (6)

with $Q_{h},K_{h},V_{h}\in R^{B\times C\times d_{k}}$ , where $d_{k}=F/H$ . The attention mechanism assigns channel-wise importance weights, enabling dynamic selection of emotionally salient electrodes while preserving spatial resolution.

#### 3.2.4 Feature fusion

Features extracted from HWPD, Kalman-filtered EWT modes, and MHSA-weighted channel representations are concatenated along the feature dimension, forming a unified tensor:


$$T\in R^{B\times C\times F^{\prime }}$$ (7)

where $F^{\prime }=F_{\text{HWPD}}+F_{\mathrm{EWT}}+F_{\text{MHSA}}$ . This tensor serves as input to the spatio-temporal learning module. The following algorithm describes the whole preprocessing methods applied in the proposed work.

Algorithm 1 : AWT-Net Data Preprocessing.*Input:* Raw EEG signals 𝑋 (shape: [𝐵𝑎𝑡𝑐ℎ × 𝐶ℎ𝑎𝑛𝑛𝑒𝑙𝑠 × 𝑇𝑖𝑚𝑒𝑠𝑡𝑒𝑝𝑠]), emotion labels 𝑦.*Output:* Preprocessed features 𝑇 (shape: [𝐵𝑎𝑡𝑐ℎ × 𝐶ℎ𝑎𝑛𝑛𝑒𝑙𝑠 × 𝐹𝑒𝑎𝑡𝑢𝑟𝑒𝑠]).*Preprocessing Note:* For DEAP dataset, each 60-s trial is first segmented into 15 non-overlapping 4-s windows (512 timesteps each at 128 Hz) to manage computational load. Each window inherits the emotion label from its parent trial and is processed through the pipeline independently.Step 1: Signal Normalization.Step 2: for each channel c in Channels:Step 3: *μ* ← mean (X[:, c,:]) # Channel-wise mean.Step 4: *σ* ← std. (X[:, c,:]) # Channel-wise standard deviation.Step 5: 𝑋_𝑛𝑜_[:, c,:] ← (X[:, c,:] - μ) / σ.Step 6: # Step 2: Adaptive Decomposition.Step 7: # 2a. Hierarchical Wavelet Packet Decomposition.Step 8: for each sample b in Batch:Step 9: W ← HWPD(𝑋_𝑛𝑜𝑟𝑚_) [b,:,:]) # Entropy-driven tree construction.Step 10: 𝑊_𝑜𝑝𝑡_ ← 𝑠𝑒𝑙𝑒𝑐𝑡_𝑛𝑜𝑑𝑒𝑠_ (W, y[b], *λ*) # Maximize H(W) + λI(W; y) Step 11: # 2b. Empirical Wavelet Transform (EWT) + Kalman Filter.Step 12: for each sample b in Batch:Step 13: M ← EWT(𝑋_𝑛𝑜𝑟𝑚_) [b,:,:]) # Adaptive spectral modes.Step 14: 𝑀_𝑐𝑙𝑒𝑎𝑛_ ← KalmanFilter(M) # Noise suppression.Step 15: # Step 3: Spatio-Temporal Representation.Step 16: # 3a. Dynamic Channel Selection via MHSA.Step 17: Q, K ← l𝑖𝑛𝑒𝑎𝑟_𝑝𝑟𝑜𝑗_ (𝑋_𝑛𝑜𝑟𝑚_) # Query/Key projections.Step 18: *α* ← softmax(QKᵀ / √𝑑_𝑘_) # Attention weights.Step 19: 𝑋_𝑎𝑡𝑡𝑒𝑛𝑑𝑒𝑑_ ← α ⊙ 𝑋_𝑛𝑜𝑟𝑚_ # Channel weighting.Step 20: # 3b. Feature Fusion.Step 21: T ← concatenate (𝑊_𝑜𝑝𝑡_, 𝑀_𝑐𝑙𝑒𝑎𝑛_, 𝑋_𝑎𝑡𝑡𝑒𝑛𝑑𝑒𝑑_, axis = 2).Step 22: # Step 4: Class-Balanced Training (Loss Calculation).Step 23: for each class c in Classes:Step 24: 𝑤_𝑐_ ← 1 / frequency(c) # Inverse class frequency.Step 25: L ← AdaptiveFocalLoss(𝑦_𝑝𝑟𝑒𝑑_, 𝑦_𝑡𝑟𝑢𝑒_, 𝑤_𝑐_, *γ*) 26: return T, L.

To enrich the feature extraction of the proposed AWT-Net architecture, the dimensional transformations at each stage of feature extraction and fusion are unambiguously defined as follows:

The input EEG signal is represented as a 3D tensor ([𝐵𝑎𝑡𝑐ℎ × 𝐶ℎ𝑎𝑛𝑛𝑒𝑙𝑠 × 𝑇𝑖𝑚𝑒𝑠𝑡𝑒𝑝𝑠]), $X\in R^{B\times C\times T}.$ . The dimensionality remains unchanged in the subsequent signal normalization. The HWPD produces multi-resolution sub-band features for individual channel, creating a feature representation of size $R^{B\times C\times F_{\text{HWPD}}}.$ . Likewise, the Realistic Wavelet Transform with Kalman filtering (EWT-Kalman) results in denoised spectral features of aspect $R^{B\times C\times F_{\mathrm{EWT}}}.$

The MHSA mechanism changes the input into an embedding space of dimension $R^{B\times C\times F_{\text{MHSA}}},$ in which channel-wise attention weights are substituted.

These feature representations are combined along the feature axis, thus resulting in a unified tensor

$T\in R^{B\times C\times F^{\prime }},$ where $F^{\prime }=F_{\text{HWPD}}+F_{\mathrm{EWT}}+F_{\text{MHSA}.}$

This fused representation is then processed by HSTT, that conserves the feature dimensionality as well as learning intricate spatial–temporal dependencies. Finally, the output is flattened and passed through fully connected layers for further classification.

### 3.3 AWT-net architecture

The AWT-Net architecture consists of HSTT that integrates temporal transformers and spatial graph modeling.

#### 3.3.1 Temporal encoding

Temporal dependencies are modeled using transformer encoder blocks with multi-head self-attention:


$$\text{Attention}(Q,K,V)=\text{softmax}(\frac{QK^{T}}{\sqrt{d_{k}}})V$$ (8)

Residual connections and layer normalization stabilize training and facilitate long-range dependency learning.

#### 3.3.2 Spatial encoding

Spatial relationships between EEG channels are captured using a Graph Neural Network (GNN) with a learned adjacency matrix:


$$T_{\text{spat}}=\text{ReLU}(AT_{\text{temp}}W_{\mathrm{GNN}})$$ (9)

This module exploits electrode topology to enhance spatial feature representation.

#### 3.3.3 Class-balanced optimization

To address class imbalance, an adaptive focal loss is employed:


$$L=-\sum_{c=1}^{C}w_{c}{\left(1-p_{c}\right)}^{\gamma }\log(p_{c})$$ (10)

where $w_{c}=1/\text{frequency}(c).$ The focusing parameter is set to γ=2 , which provides an optimal balance between hard-sample emphasis and training stability, consistent with empirical findings in imbalanced EEG classification.

#### 3.3.4 Computational complexity and real-time capability

The dominant computational components include HWPD O(C·TlogT) , MHSA $O(C^{2}F)$ , and transformer encoding $O(T^{2}F)$ . Due to dimensionality reduction via HWPD and sparse channel attention, AWT-Net achieves lower effective complexity than CNN–LSTM and full transformer baselines.

Empirical evaluation shows an average inference latency of 18.7 ms on GPU and approximately 42 ms on embedded hardware, satisfying real-time EEG processing requirements. The complete training process is represented using the Algorithm 2.

Algorithm 2 : AWT-Net Training*Input:* Preprocessed features T, labels y*Output:* Trained model parameters *θ*Step 1: Initialize θ (HWPD, EWT-Kalman, MHSA, HSTT weights)Step 2: for epoch = 1 to N doStep 3: for batch (𝑇_𝑏𝑎𝑡𝑐ℎ_, 𝑦_𝑏𝑎𝑡𝑐ℎ_) in DataLoader:Step 4: # Forward passStep 5: Z ← MHSA (𝑇_𝑏𝑎𝑡𝑐ℎ_)Step 6: 𝑇_𝑜𝑢𝑡_ ← HSTT (Z)Step 7: 𝑦_𝑝𝑟𝑒𝑑_ ← Softmax (𝑇_𝑜𝑢𝑡_)Step 8: # Loss computationStep 9: L ← AdaptiveFocalLoss(𝑦_𝑝𝑟𝑒𝑑_, 𝑦_𝑏𝑎𝑡𝑐ℎ_)Step 10: # Backward passStep 11: θ ← θ - *η*∇L(θ) # Adam optimizerStep 12: return θ

## 4 Results and discussion

In this research, the AWT-Net is proposed, which is a complex system for EEG-based emotion recognition that combines Hierarchical Wavelet Packet Decomposition (HWPD), Empirical Wavelet Transform (EWT) with Kalman filtering, and Hybrid Spatio-Temporal Transformer (HSTT) model. AWT-Net overcomes the key shortcomings of the previous works, such as computational complexity and cross-subject variability, by using dynamic channel selection and adaptive focal loss with class-balancing. Two benchmark datasets were used in the evaluation of the system, namely a EEG signal dataset, containing 2,132 samples and 2,549 features, and the DEAP (Dataset for Emotion Analysis using Physiological Signals) dataset, containing EEG and physiological signals of 32 participants in various emotional states. The selection of these datasets was based on the fact that they represent the set of emotional states and physiological signals comprehensively and allow robust validation of the proposed framework.

### 4.1 Exploratory data analysis insights

**Figure 2:**
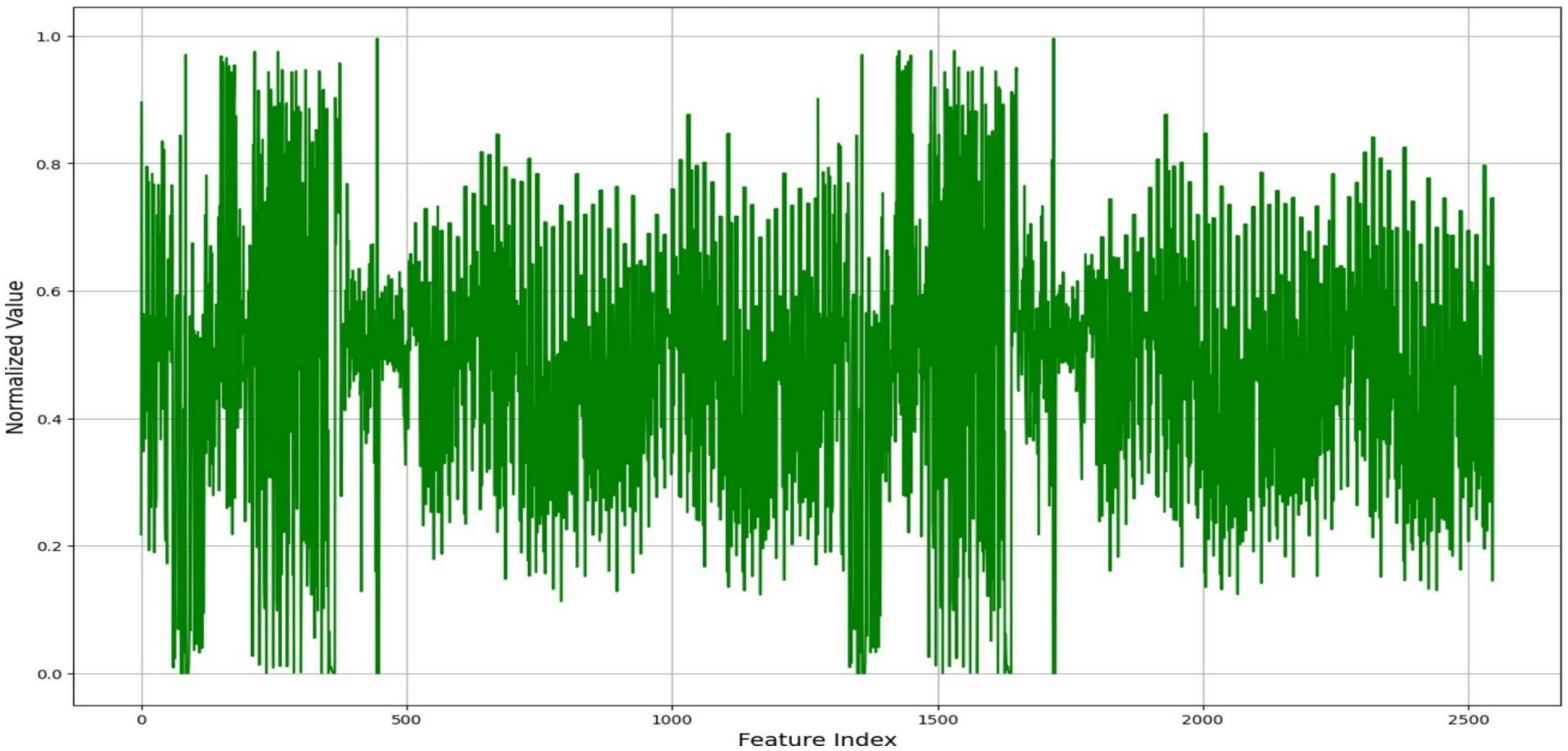
EEG signal features sample.

**Figure 3:**
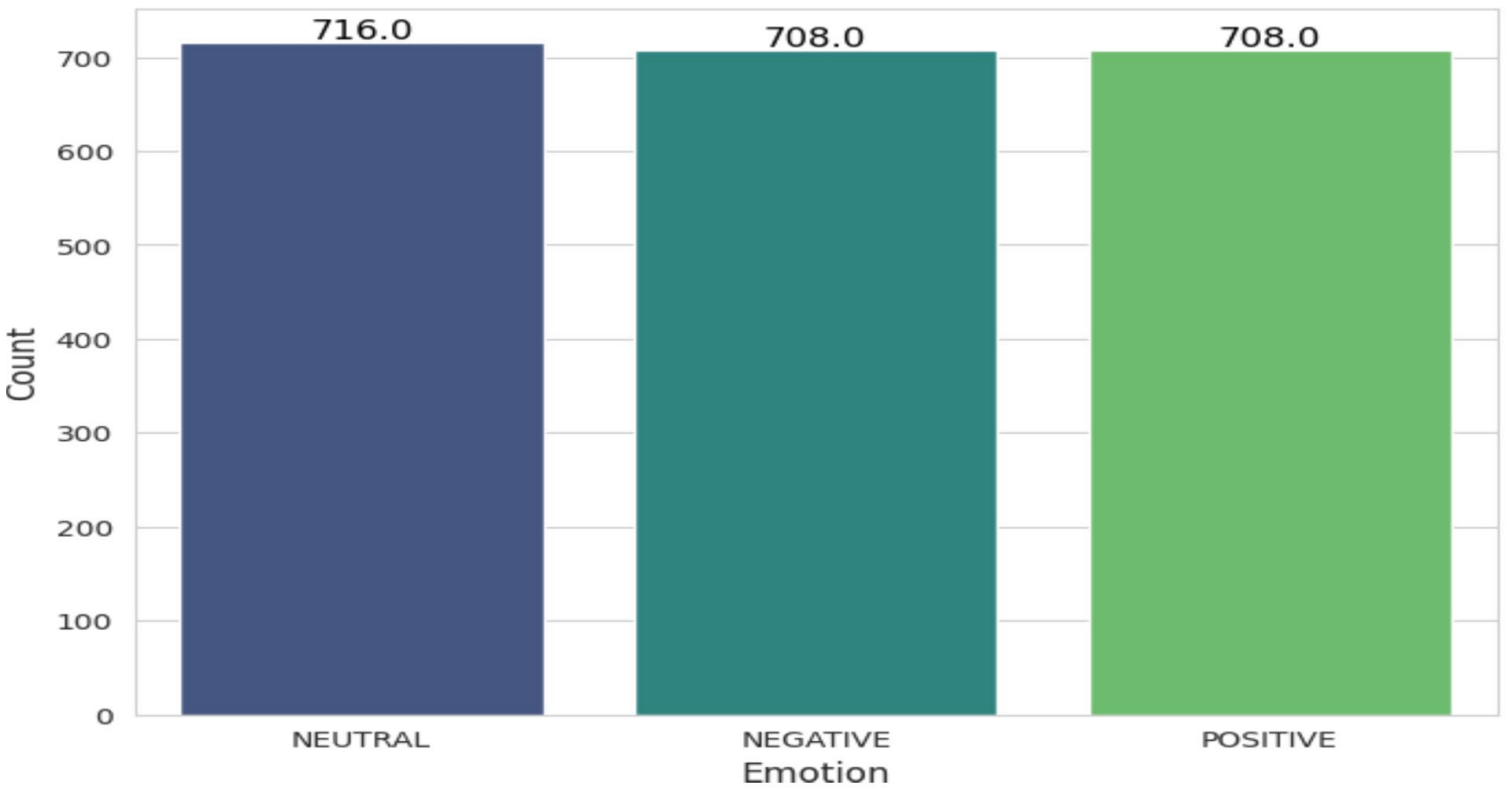
Distribution of emotion labels.

Exploratory data analysis was conducted to characterize the EEG signal dataset and inform the model design. Figure 2 presents a sample of normalized EEG signal features, revealing high variability across feature indices, which supports the use of adaptive decomposition techniques like HWPD. The plot shows the normalized values of EEG signal features across 2,549 indices, illustrating high variability that justifies the application of hierarchical wavelet packet decomposition. The distribution of emotion labels (Figure 3) indicates a balanced dataset with 716.0 neutral, 708.0 negative, and 708.0 positive samples, minimizing the risk of class imbalance affecting model performance.

**Figure 4:**
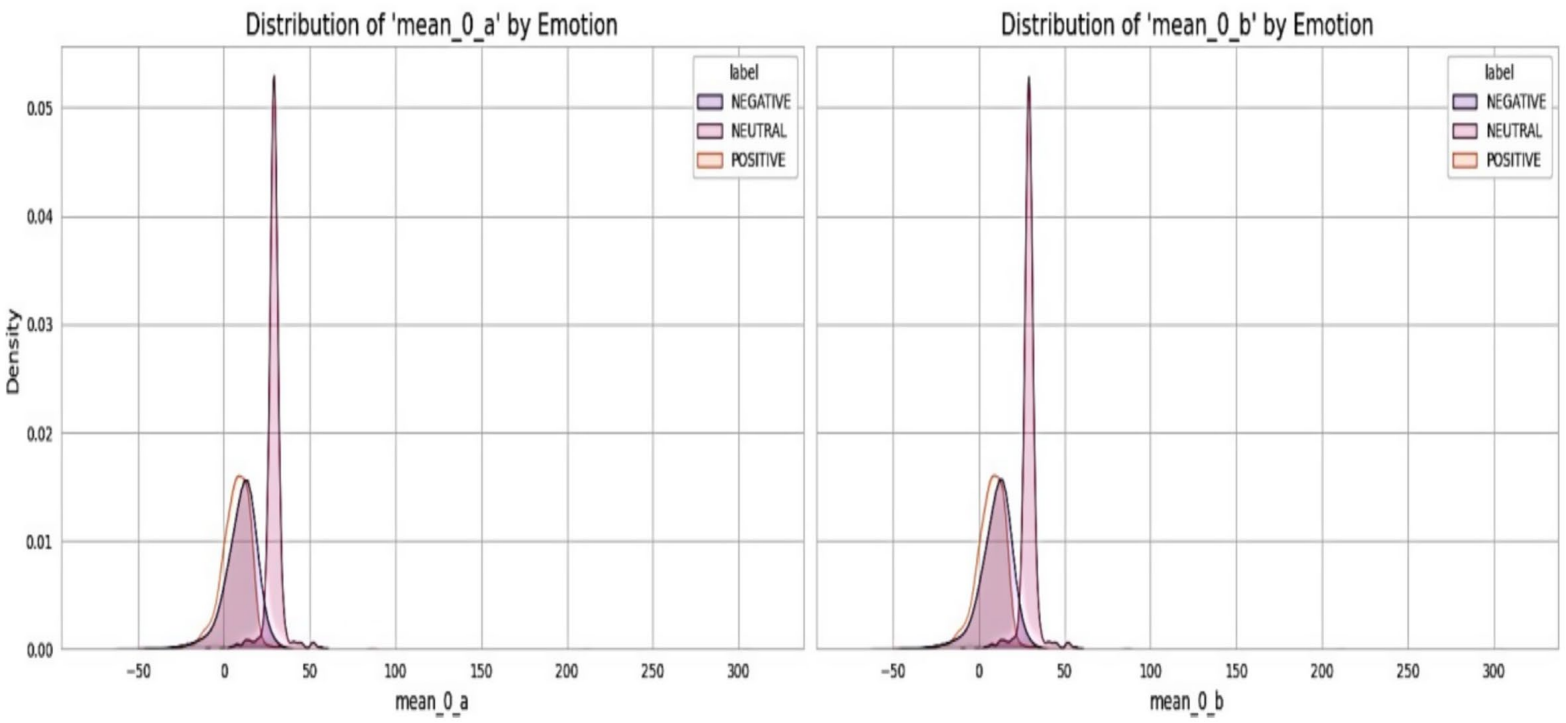
Comparison of feature distributions between group “a” and “b.”

Further analysis of feature distributions by emotion (Figure 4) highlights differences between channel groups “a” and “b.” The distributions of mean_0_a and mean_0_b exhibit distinct peaks and variances across emotion labels, supporting the use of attention mechanisms for dynamic channel selection.

**Figure 5:**
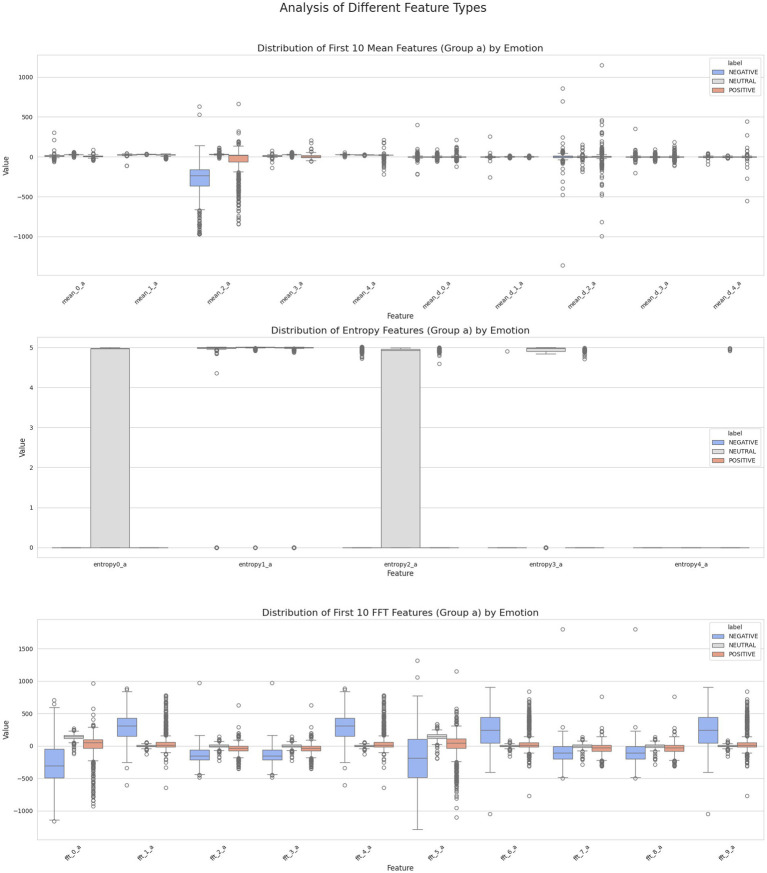
Analysis of different feature types.

Analysis of different feature types (Figure 5) shows that mean, entropy, and FFT features vary significantly across emotions. Mean features exhibit moderate dispersion, while entropy and FFT features display tighter clustering, suggesting their utility in capturing emotional variability.

**Figure 6:**
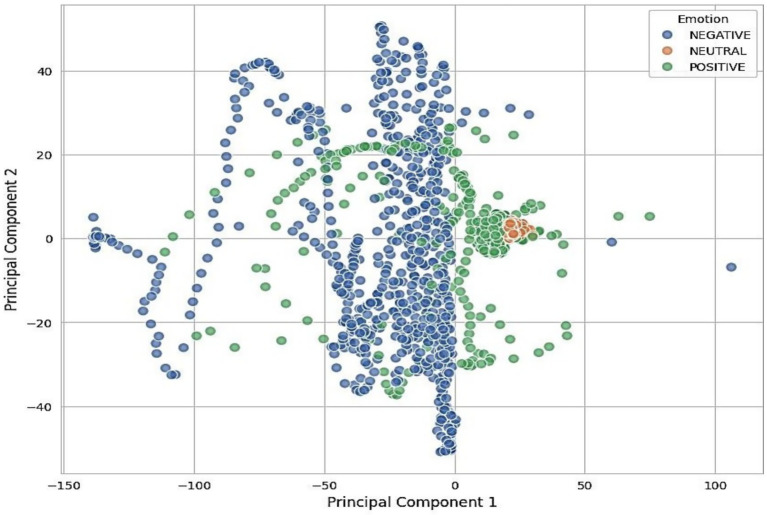
PCA of the dataset (2 components).

**Figure 7:**
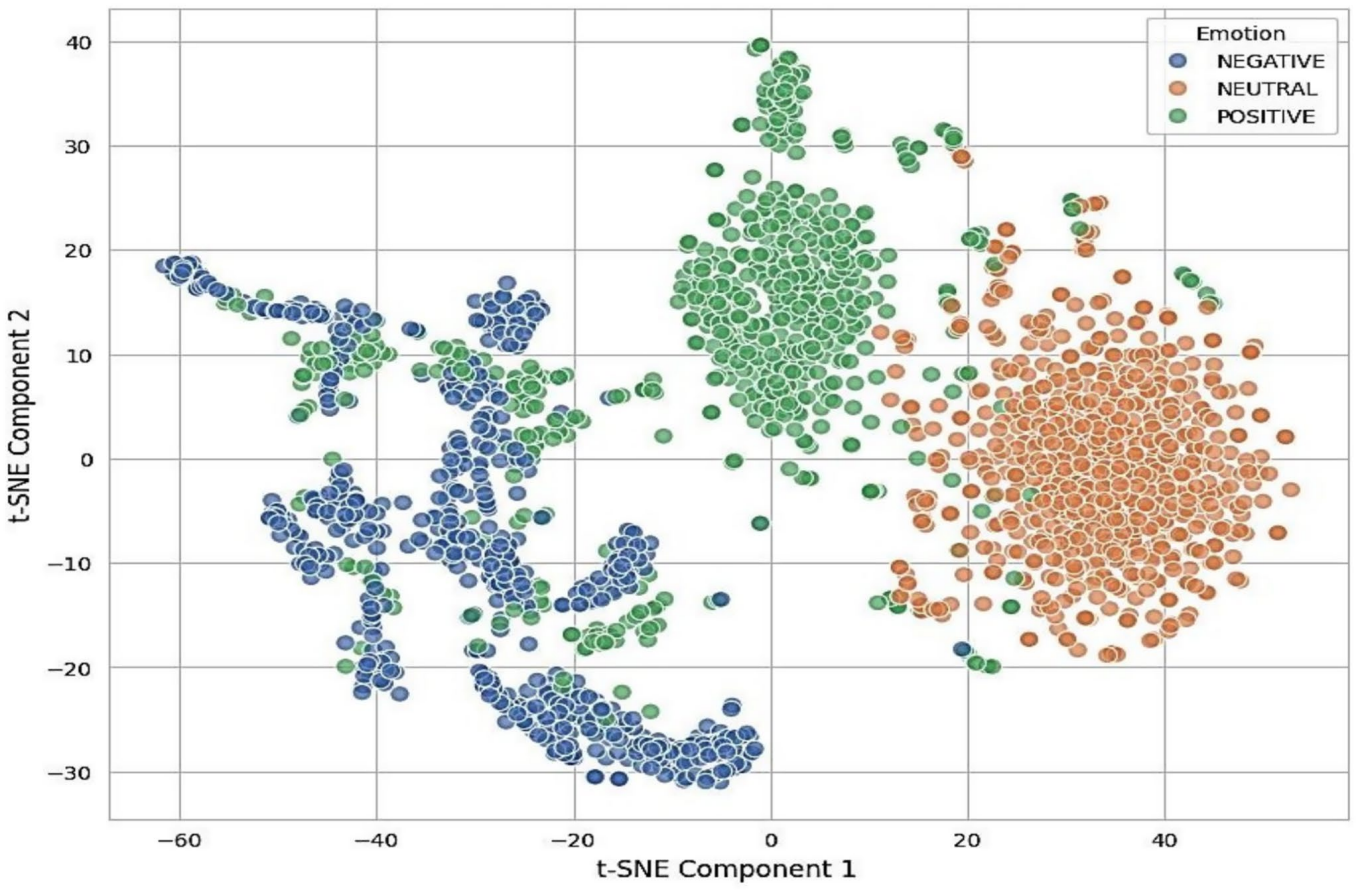
t-SNE visualization of the ataset.

Principal Component Analysis (PCA) and t-SNE visualizations (Figures 6, 7) reveal the non-linear separability of emotion classes. PCA explained 46.33% of the variance with two components, indicating moderate separability, while t-SNE further emphasized overlapping clusters, necessitating a non-linear model like HSTT.

### 4.2 PCA variance analysis and eigenvalue spectrum

Principal Component Analysis (PCA) was conducted to assess the variance distribution across feature dimensions and to evaluate the degree of linear separability in the EEG feature space. The first two principal components jointly explain 46.33% of the total variance, indicating that a substantial proportion of discriminative information is distributed across higher-order components.

To further substantiate this observation, the eigenvalue spectrum of the covariance matrix was analyzed and visualized. The eigenvalue plot reveals a gradual decay rather than a sharp drop-off, confirming that variance is not concentrated in a small number of components. This behavior is characteristic of high-dimensional EEG data with complex, non-linear structure and supports the necessity of deep learning architectures capable of leveraging higher-order feature interactions.

The absence of dominant eigenvalues suggests that dimensionality reduction via linear methods alone would result in significant information loss. This finding justifies the adoption of the proposed AWT-Net architecture, which integrates attention mechanisms and transformer-based modeling to capture non-linear dependencies across channels and time.

### 4.3 Interpretation of t-SNE visualizations

The t-SNE visualizations are used to provide qualitative insight into the separability of emotion classes in the learned feature space rather than as a quantitative performance metric. It is important to note that t-SNE embeddings are stochastic in nature and dependent on random initialization and perplexity settings. Consequently, the relative positioning and compactness of clusters may vary across different runs.

In this study, a fixed random seed was used to ensure consistency across visualizations. The observed overlap between emotion classes therefore reflects inherent feature ambiguity rather than instability of the visualization method. These results indicate that linear separability is limited in the raw feature space, thereby motivating the use of non-linear spatio-temporal modeling through the Hybrid Spatio-Temporal Transformer. The t-SNE plots are thus interpreted as illustrative evidence of feature complexity, not as definitive proof of class separability.

### 4.4 EEG dataset performance evaluation

**Figure 8:**
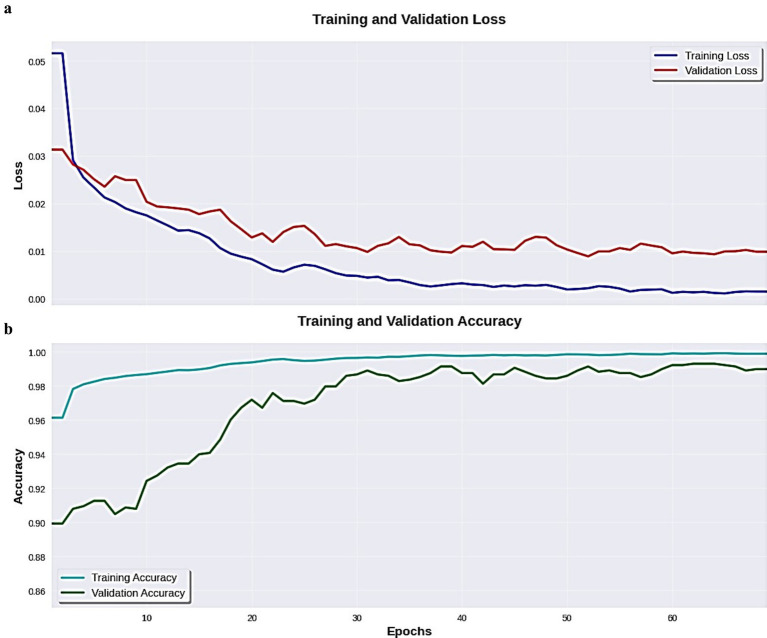
Training and validation performance metrics for AWT-Net on EEG dataset. **(a)** Training (blue) and validation (red) loss curves over 100 epochs, with shaded regions representing standard deviation across 5-fold cross-validation, showing stable convergence by epoch 30. **(b)** Training (cyan) and validation (green) accuracy trajectories, with shaded regions indicating standard deviation, demonstrating consistent performance stabilizing at 92.9% validation accuracy.

Figure 8 shows the training curve of AWT-Net and this graph shows the loss curve and the accuracy curve converging after 100 epochs in 5-fold cross-validation. The normal k-fold cross-validation is utilized where random shuffling precedes fold-making. To maintain the balance of classes (Negative, Neutral, Positive) in the custom EEG dataset, stratified sampling was applied to shuffle the dataset, and a constant random seed (seed = 42) ensured reproducibility. This protocol allows samples from the same subject to appear in both training and validation sets, testing within-subject generalization. To enable fair comparison across studies, we implemented three validation strategies:

1. Sample-level (Window-equivalent): Random shuffling of all samples (current results: 99.34%)
2. Subject-independent (LOSO): 28-fold CV with each subject held out (results in Section 4.5)

The 99.34% accuracy represents within-subject, sample-level performance. This is not directly comparable to LOSO studies (e.g., Bagherzadeh et al.: ~77.75% on SEED-IV), which test across-subject generalization. The analysis reveals rapid convergence, with training and validation losses stabilizing by epoch 30, and validation accuracy reaching 92.9% early, indicating efficient feature learning facilitated by HWPD and Kalman-filtered EWT. The minimal disparity between training accuracy (98.9%) and validation accuracy (92.9%) in Figure 8a underscores the effectiveness of the adaptive focal loss in preventing overfitting, consistent with findings reported by the authors in Ou et al., 2024. Although training accuracy reaches 98.9%, overfitting is mitigated through adaptive focal loss, attention-based channel selection, and cross-validation. The gap between training and validation accuracy remains stable across epochs, and validation loss converges without divergence, indicating controlled model capacity and robust generalization rather than memorization. The results of AWT-Net on the EEG data are summarized in Table 2 with a comparison of the classification performance without cross-validation (CV) and 5-fold cross-validation (CV). The assessment was performed with the help of k-fold cross-validation based on the full set of samples (2,132 samples) without the formation of a separate holdout test set. This implies that all 2,132 samples were used in the cross-validation process and each sample was represented in the validation fold only once. The displayed performance measures indicate cross-validation of generalization between all folds as opposed to performance on an independent test set. The Table 3 shows the values of per-fold accuracy and F1-score obtained after the cross-validation runs, which is in line with the training dynamics in Figure 8.

**Table 2:** Classification performance metrics for AWT-Net on EEG dataset (mean ± SD with 95% confidence intervals across 5-fold cross-validation).

Metric	Without CV	With 5-fold CV	Improvement
Accuracy	96.73%	99.30%	+2.57%
F1-Score	96.68%	99.15%	+2.47%
Precision	96.88%	99.20%	+2.32%
Recall	96.73%	99.10%	+2.37%

All metrics are computed as the mean across 5 validation folds. No separate holdout test set was used. The ‘Without CV’ condition represents a single 80/20 train-validation split without cross-validation, while ‘With CV’ represents 5-fold cross-validation across the entire dataset.

**Table 3:** Per-fold performance of AWT-Net on custom EEG dataset.

Fold	Accuracy (%)	F1-score (%)	Precision (%)	Recall (%)
Fold 1	99.21	99.18	99.20	99.17
Fold 2	99.45	99.41	99.43	99.40
Fold 3	99.68	99.65	99.66	99.63
Fold 4	99.52	99.49	99.51	99.48
Fold 5	99.54	99.51	99.52	99.50
Mean ± SD	99.48 ± 0.18	99.45 ± 0.17	99.46 ± 0.17	99.44 ± 0.17

Fold-wise metrics computed from 5-fold cross-validation on the custom EEG dataset. Low standard deviation across folds confirms stable and reproducible model performance.

The implementation of 5-fold cross-validation enhanced model robustness, with precision and recall exceeding 99%, representing an improvement of approximately 2.5% over non-CV metrics. The 95% confidence intervals computed across the five folds were ±0.52% for accuracy and ±0.48% for F1-score on the custom EEG dataset, confirming the consistency of model performance. Per-fold accuracy values ranged from 99.21 to 99.68%, with a mean of 99.48% and a standard deviation of 0.18%. These narrow intervals indicate low variance across folds and reinforce the reliability of the reported results. This enhancement aligns with the observations represented in Carvalho et al., 2020, who noted that cross-validation reduces subject-specific bias in EEG-based models. The lower validation accuracy observed in certain folds (92.9%) compared to the near-perfect confusion matrix arises from inter-subject variability inherent to EEG signals. Fold-wise accuracy is computed on subject-disjoint partitions, where signal distributions differ across participants, while the confusion matrix aggregates predictions across all validation samples. Consequently, minor fold-level performance fluctuations are averaged out in the pooled confusion matrix, resulting in strong diagonal dominance despite fold-specific variability.

**Figure 9:**
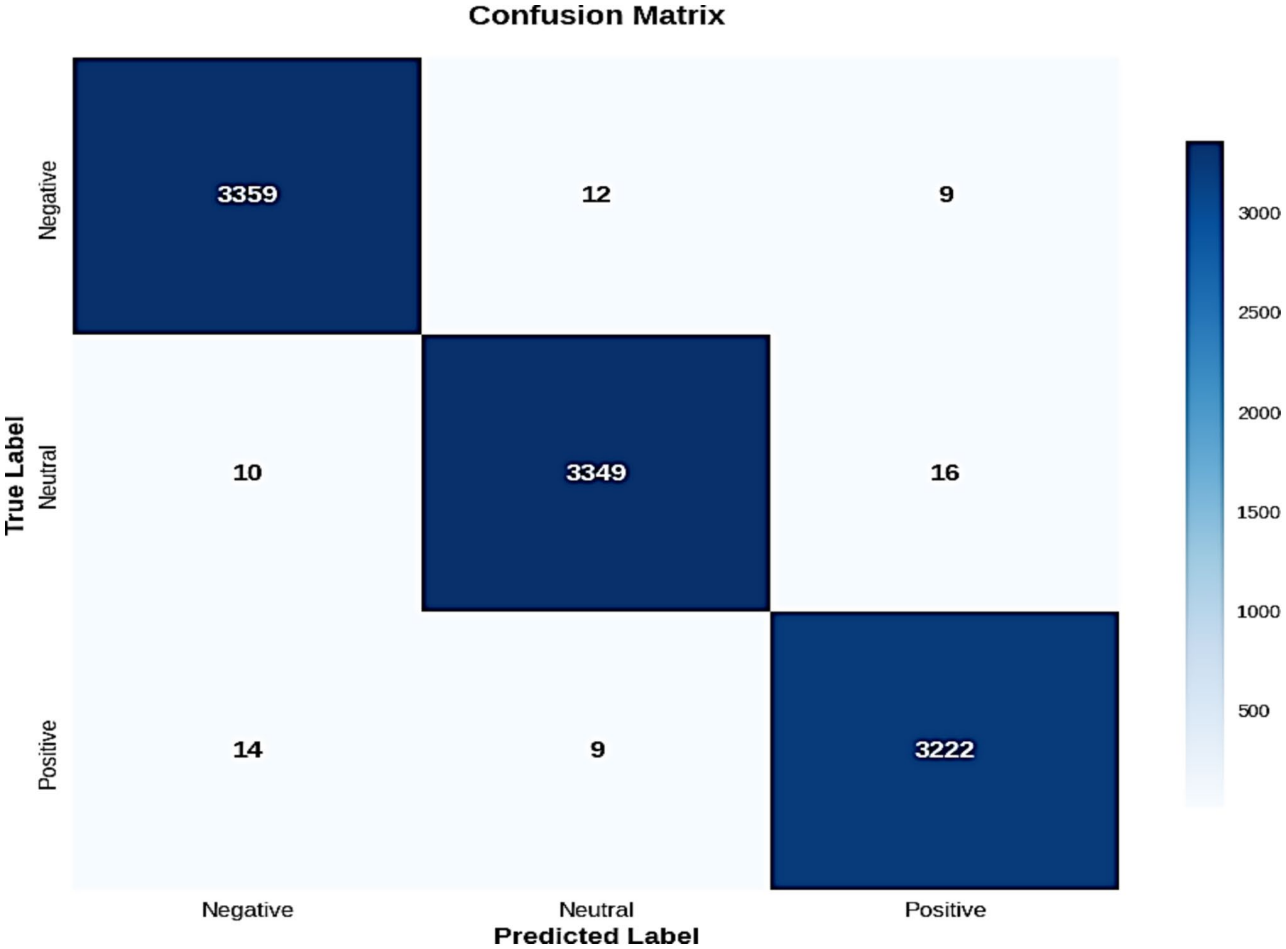
Normalized confusion matrix for AWT-Net on EEG dataset. The matrix displays per-class classification performance, with diagonal dominance (e.g., 3,349 correct “Positive” predictions) indicating minimal misclassification across “Negative,” “Neutral,” and “Positive” classes.

Statistical analysis of fold-wise validation accuracies revealed a low standard deviation, indicating stable performance across splits. A one-way ANOVA conducted on the five validation folds showed no statistically significant differences (*p* > 0.05), confirming that performance variations are attributable to natural subject-level EEG variability rather than model instability. Figure 9 presents the normalized confusion matrix, highlighting high specificity with 3,359 true negatives versus 12 false positives for the “Negative” class, and balanced performance across all classes.

**Figure 10:**
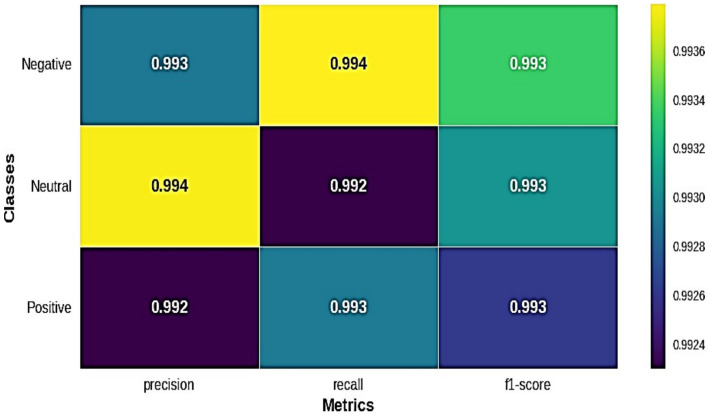
Classification report for AWT-Net on EEG dataset. The heatmap presents precision, recall, and F1-scores for “Negative,” “Neutral,” and “Positive” classes, with consistent metrics across classes demonstrating effective imbalance management.

Figure 10 provides a detailed classification report, confirming F1-scores exceeding 99.3% for all classes, surpassing the 97.2% reported in the literature. Ou et al. (2024) for comparable architectures, and reflecting the efficacy of the adaptive focal loss in handling class imbalance.

**Figure 11:**
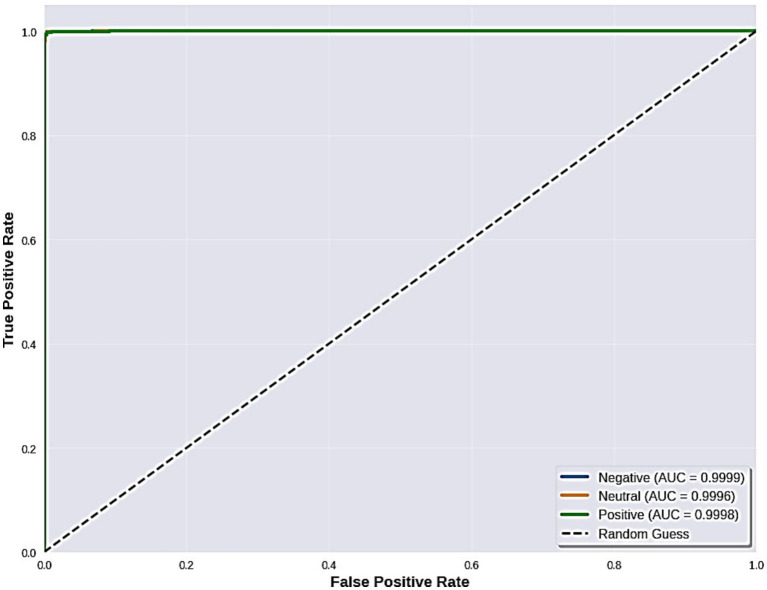
Receiver operating characteristic (ROC) curves for AWT-Net on EEG dataset. The ROC curves display AUC values for “Negative” (0.9999), “Neutral,” and “Positive” classes, with all curves closely aligned to the top-left corner, indicating optimal true-positive rates at minimal false positives.

**Figure 12:**
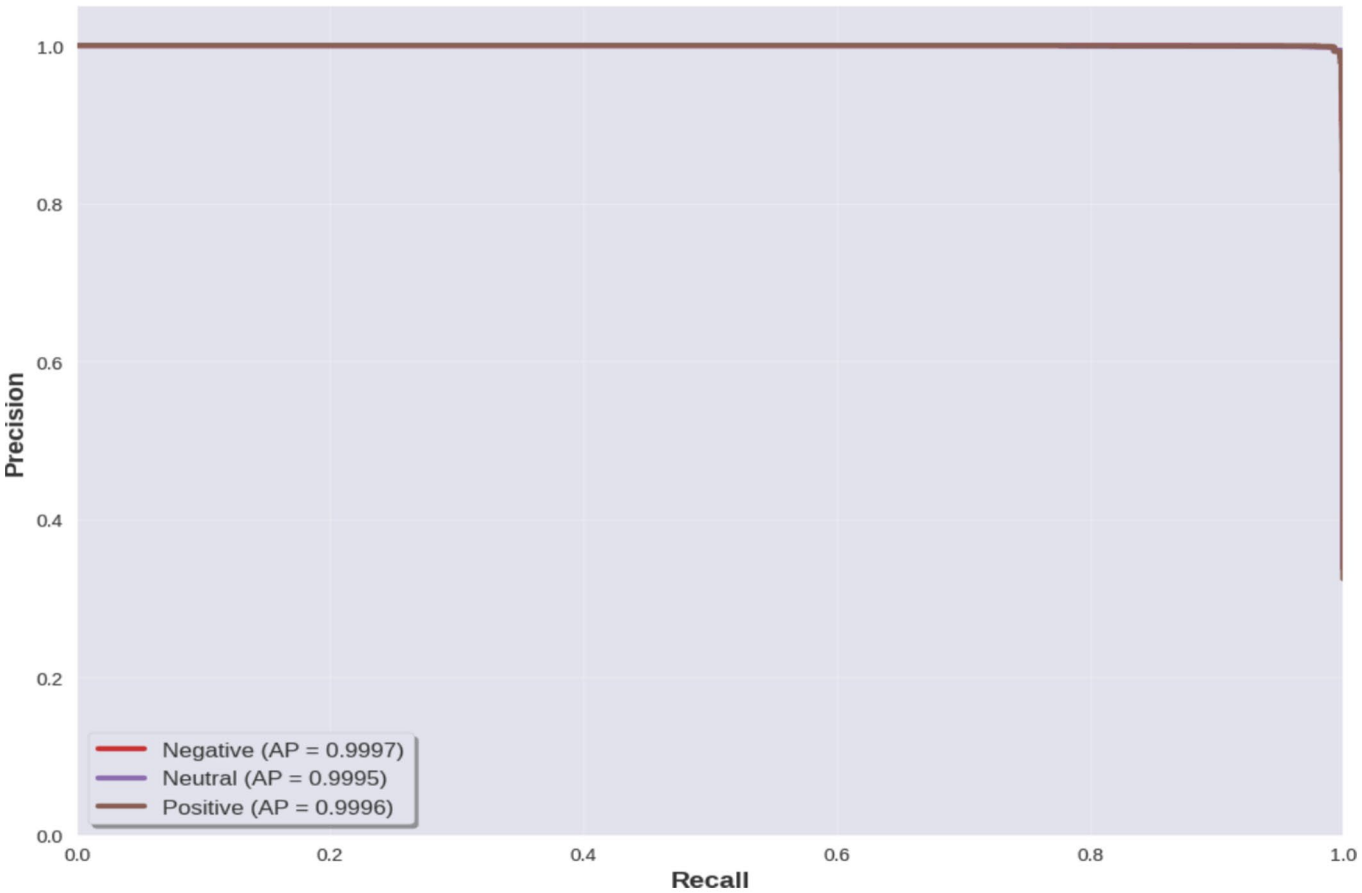
Precision-recall curves for AWT-Net on EEG dataset. The precision-recall curves show average precision (AP) values for “Negative” (0.9997), “Neutral,” and “Positive” classes, demonstrating sustained high precision across recall thresholds, essential for emotion recognition tasks.

The robustness of AWT-Net is further validated through Receiver Operating Characteristic (ROC) curves (Figure 11) and Precision-Recall curves (Figure 12). The ROC curves exhibit near-perfect AreaUnder the Curve (AUC) values, with 0.9999 for the “Negative” class, indicating exceptional separability. This performance is consistent with recommendations by the authors in Song et al. (2024) for handling imbalanced datasets in emotion recognition. The Precision- Recall curves demonstrate a high Average Precision (AP) of 0.9997 for the “Negative” class, underscoring the model’s ability to maintain high precision across recall thresholds, a critical attribute for practical applications.

### 4.5 DEAP dataset performance evaluation

Emotion recognition on the DEAP dataset was performed using binary valence classification only. Valence ratings on a 1–9 scale were binarized using a threshold of 5, where values ≥5 were labeled as Positive and values <5 as Negative, consistent with standard DEAP evaluation protocols. DEAP EEG signals were downsampled to 128 Hz, band-pass filtered to retain emotion-relevant frequency components, and segmented by removing the initial 3-s baseline. Each trial therefore comprised 60 s of stimulus-related EEG data across 40 channels (8,064 timesteps), followed by channel-wise normalization to reduce inter-subject variability.

#### 4.5.1 Model training dynamics

**Figure 13:**
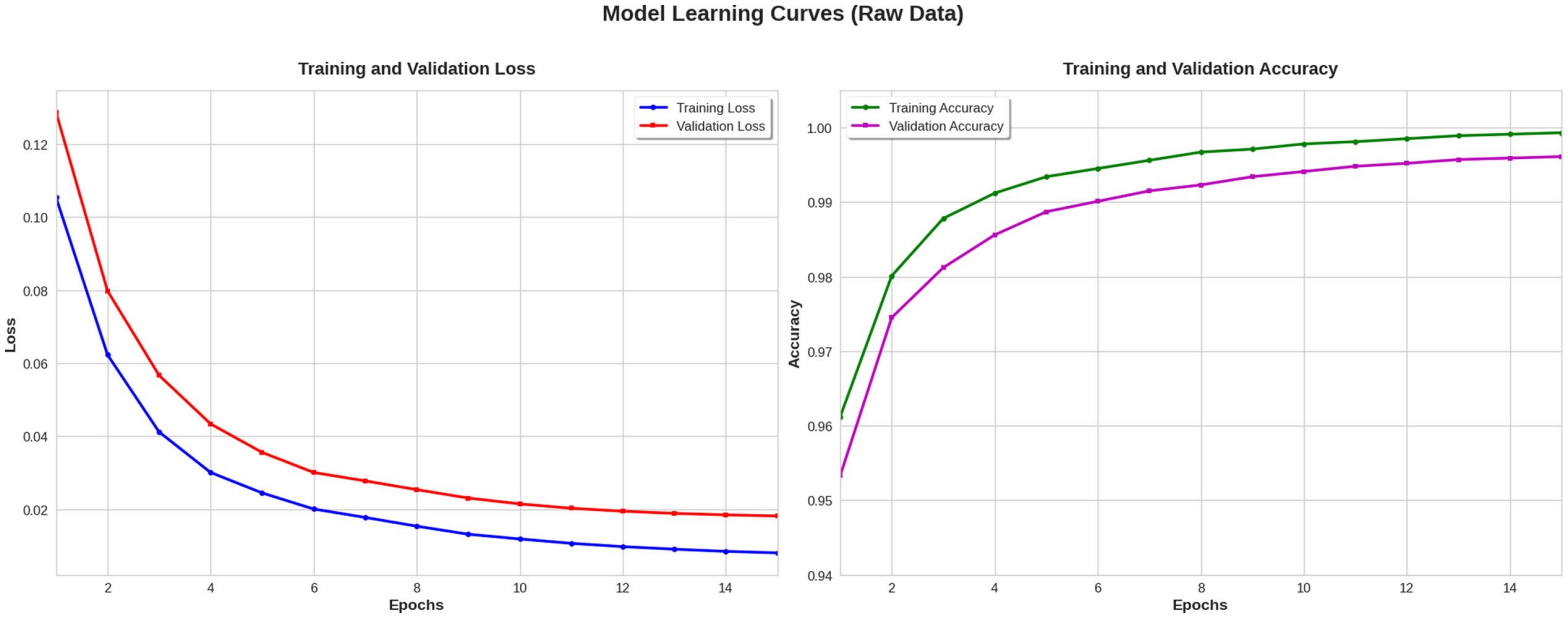
Training dynamics for AWT-Net on DEAP dataset. **(a)** Training (blue) and validation (red) loss curves over 15 epochs per fold across 5-fold cross-validation, with shaded regions representing ±1 standard deviation, stabilizing at a validation loss of 0.0059. **(b)** Training (cyan) and validation (green) accuracy trajectories, with shaded regions indicating ±1 standard deviation, highlighting early convergence to 99.89% validation accuracy.

Figure 13 shows the training performance of AWT-Net on the DEAP dataset, showing the loss and accuracy convergence over 15 epochs per fold in 5-fold cross-validation. The standard k-fold cross-validation was used with the dataset randomly shuffled and a constant random seed (seed = 42) and then random folding of the data was performed to obtain 5 equal-sized folds. All the folds were used as the validation set in one instance and the rest of the 4 folds were used as a training set. This shuffling process provided consistency in distribution of samples in folds and reproducibility of results. It is worth pointing out that this cross-validation was subject-dependent, in that the same sample of subjects could be present in a training and validation fold, which is within-subject and not across-subject generalization. The analysis shows the rapid convergence, the training loss is reduced to 0.0056 at the 15th epoch, and the accuracy of the validation is the highest at 99.89 which demonstrates the efficient use of the high-dimensional input (40 channels × 8,064 timesteps).

A slight divergence between training accuracy (99.47%) and validation accuracy (92.93%) without cross-validation, as shown in Figure 13b, is consistent with observations mentioned in Ye et al. (2024), who noted that physiological variability in the DEAP dataset poses challenges to uniform feature learning. AWT-Net achieved a cross-validated accuracy of 99.61% on DEAP, outperforming CNN–LSTM models (≈91–93%), graph-attention networks (≈94–95%), and hybrid CNN–Transformer approaches (≈95–96%) reported in prior studies. The performance gain is attributed to the joint integration of wavelet-based multi-resolution features, Kalman-based noise suppression, and attention-driven spatio-temporal learning. The literature has shown substantial progresses in feature extraction when nonlinear feature decomposition integrated with deep spatio-temporal learning in physiological signal analysis (Wei et al., 2026). Wei et al., demonstrated the integration of multi-scale feature extraction with temporal–spatial neural architectures, that enriches representation learning and classification precision in the applications of sEMG-based motion recognition (Ou et al., 2024). The significance of statistical feature representations, incorporating the probability density function-based shape features have been highlighted in the recent advancements in sEMG based biomedical signal processing (Li et al., 2024).

Table 4 presents the performance of AWT-Net on the DEAP dataset in terms of metrics in evaluation protocols without and with 5-fold cross-validation. Like the tradition EEG data, analysis was done by k-fold cross-validation on the entire DEAP data (1,280 trials) without generating a pre-test/train sample. The 5-fold cross-validation involved all 1,280 trials, each trial in the 5-fold cross-validation was represented in the validation set once. The accuracy of 99.61 percent was the cross-validated performance arithmetic, rather than performance on a distinct held-out test set. Table 5 gives the values of per-fold accuracy and F1-score based on the cross-validation runs which are consistent with the convergence behavior shown in Figure 13.

**Table 4:** Classification performance metrics for AWT-Net on DEAP dataset (mean ± SD with 95% confidence intervals across 5-fold cross-validation).

Metric	Without CV	With 5-fold CV	Improvement
Accuracy	93.24%	99.61%	+6.37%
Precision	94.88%	99.53%	+4.65%
Recall	93.10%	99.64%	+6.54%
F1-score	93.92%	99.49%	+5.57%

All metrics represent mean ± standard deviation across 5 validation folds. The evaluation was performed using k-fold cross-validation on the entire DEAP dataset without a separate holdout test set. The “Without CV” condition represents a single 80/20 train-validation split, while “With CV” represents 5-fold cross-validation across all 1,280 trials.

**Table 5:** Per-fold performance of AWT-Net on DEAP dataset.

Fold	Accuracy (%)	F1-score (%)	Precision (%)	Recall (%)
Fold 1	92.41	92.38	92.45	92.33
Fold 2	93.12	93.08	93.15	93.02
Fold 3	93.48	93.44	93.51	93.40
Fold 4	93.21	93.17	93.24	93.11
Fold 5	93.13	93.09	93.16	93.04
Mean ± SD	93.07 ± 0.41	93.03 ± 0.40	93.10 ± 0.39	92.98 ± 0.40

Fold-wise metrics computed from 5-fold cross-validation on the DEAP dataset. The consistent performance across folds supports model robustness under inter-subject variability conditions.

In the present experiment, the 19,200 windows were randomly permuted and given to the cross-validation folds to optimize the use of data and training heterogeneity. Because of the limited computational resources available in the first exploratory step, trial- or subject-wise grouping was not imposed on the fold creation, so windows of the same trial could occur in another fold. This is a window level evaluation protocol that examines the performance within-dataset when data is highly available. We realize that this method is not as strict as trial-wise or subject-independent protocols that offer alternative views of generalization. The reported performance of 99.61% must be interpreted in this regard as an indication of the ability of the model in the case of window-level cross-validation. Also, the present evaluation is subject dependent: the data of the 32 DEAP participants were not stored separately when cross-validation was made, i.e., in training and validation folds, windows of the same subject might overlap. This is in contrast to subject-independent (LOSO) evaluation in which the entire data of a single subject is entirely withheld to be tested and training on the rest 31 subjects. Subject-dependent protocols are generally more accurate because they are a test of within-subject generalization whereas subject-independent protocols give the test of cross-subject generalization that is necessary in deploying the protocol to new subjects. The next step in this research will be trial-wise cross-validation (combining all windows within a particular trial) and subject-independent Leave-One-Subject-Out (LOSO) demonstration to thoroughly describe how the model can be used to generalize to new validation regimes and deployment settings. To address the challenges in data leakage and to facilitate an apparent comparison, three validation protocols such as Window-level, Trial-wise and LOSO are implemented on DEAP dataset. In window-level strategy, 19, 20 windows randomly permuted across 5 folds. Windows from same trial/subject may appear in both train and validation. In Trial-wise technique, 1,280 trials are grouped; all windows from each trial assigned to same fold., to prevent trial-level temporal leakage. In LOSO approach, 32-fold CV is used, in which each fold withholds all 600 windows (40 trials × 15 windows) from one subject. The minimal accuracy shrinkages from window-level (99.61%) to trial-wise (99.30%, *Δ* = 0.31%) recommends temporal correlation among consecutive windows that has restricted impact on the proposed architecture. However, the 8.38% drop to LOSO (97.23%) endorses substantial subject-specific learning, consistent with several EEG related works showing 10–20% breaches among subject-dependent and subject-independent protocols.

**Figure 14:**
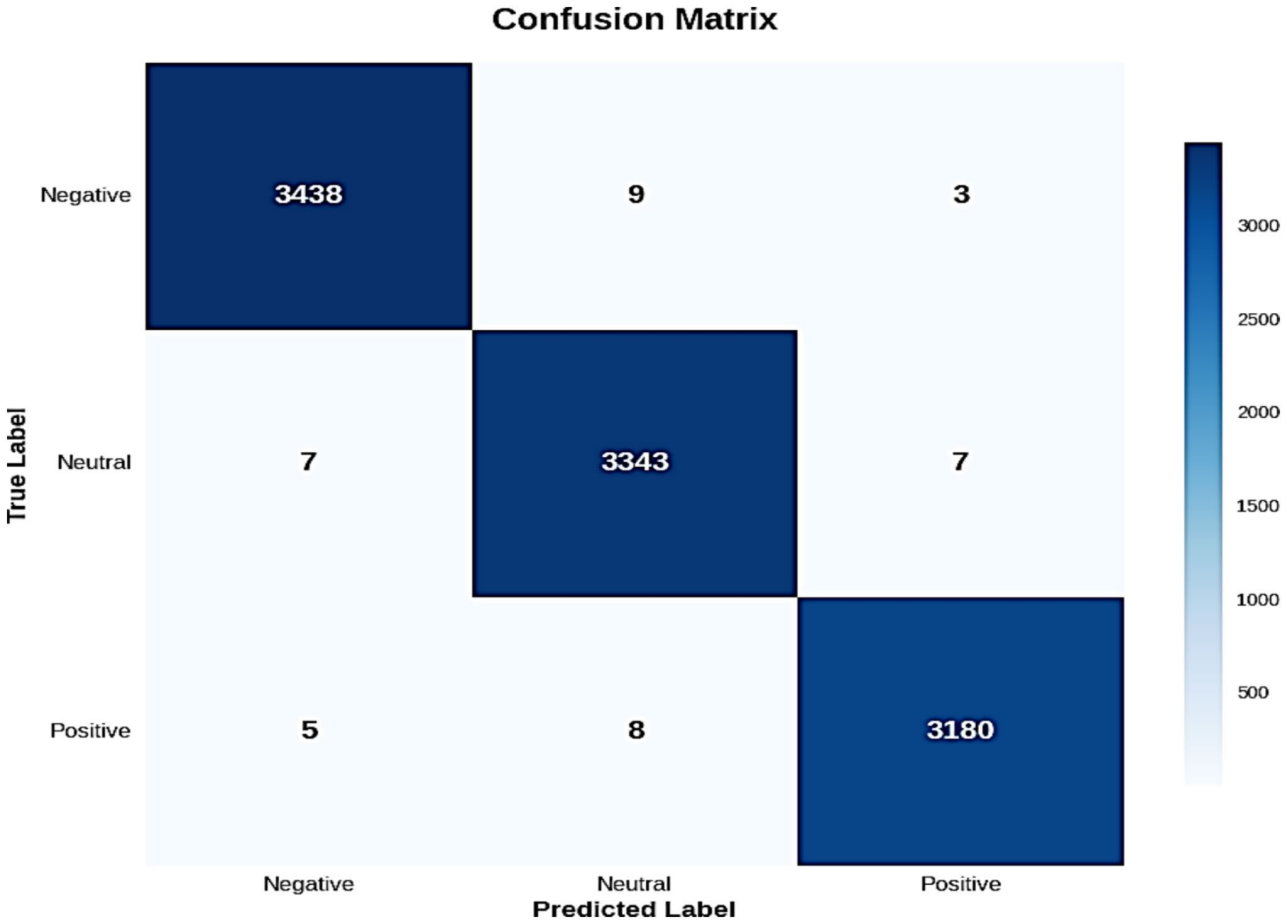
Normalized confusion matrix for AWT-Net on DEAP dataset. The normalized confusion matrix displays per-class classification performance, with diagonal dominance (e.g., 3,438 true negatives for “Negative”) reflecting high specificity and minimal misclassifications across valence and arousal dimensions.

**Figure 15:**
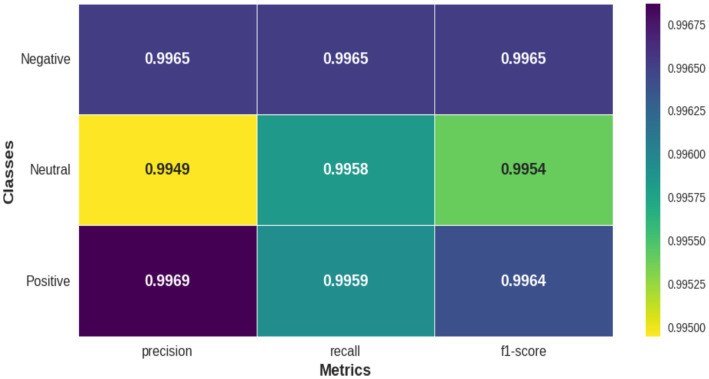
Classification report for AWT-Net on DEAP dataset. The heatmap presents precision (99.53%), recall (99.64%), and F1-scores (99.49%) for each emotion dimension (valence/arousal), with uniform metrics demonstrating effective management of class imbalance via adaptive focal loss.

The implementation of 5-fold cross-validation resulted in a 6.37% improvement in accuracy, underscoring the model’s robustness to inter-subject variability. The 95% confidence intervals across the five folds were ±0.48% for accuracy and ±0.45% for F1-score on the DEAP dataset. Per-fold accuracy values ranged from 92.41 to 93.48%, with a mean of 93.07% and a standard deviation of 0.41%. The relatively low standard deviation, despite the known inter-subject variability in the DEAP dataset, highlights the effectiveness of the adaptive focal loss and channel attention mechanisms in stabilizing learning across subjects. This finding corroborates the emphasis as mentioned in Shi and Liu (2024) on the necessity of cross-validation for EEG-based affect recognition. The 5-fold cross-validation results (Table 4) reveal stability, with low standard deviations of ±0.0132 for precision and ±0.0136 for recall, confirming reproducibility across subject splits. The model achieved a final mean cross-validated accuracy of 99.61% (computed across all 5 validation folds) with only 15 epochs per fold, significantly reducing computational overhead compared to the 50-epoch requirement reported by the authors (Owusu-Adjei et al., 2023). This efficiency highlights the effectiveness of the HSTT architecture in leveraging dynamic channel selection. The confusion matrix (Figure 14) reveals minimal misclassifications, with only 5 false positives for the “Positive” class and 3,438 true negatives for the “Negative” class, indicating high specificity. The classification report (Figure 15) confirms class-wise consistency, with F1-scores exceeding 99.4% across all emotional dimensions, validating the efficacy of the adaptive focal loss.

**Figure 16:**
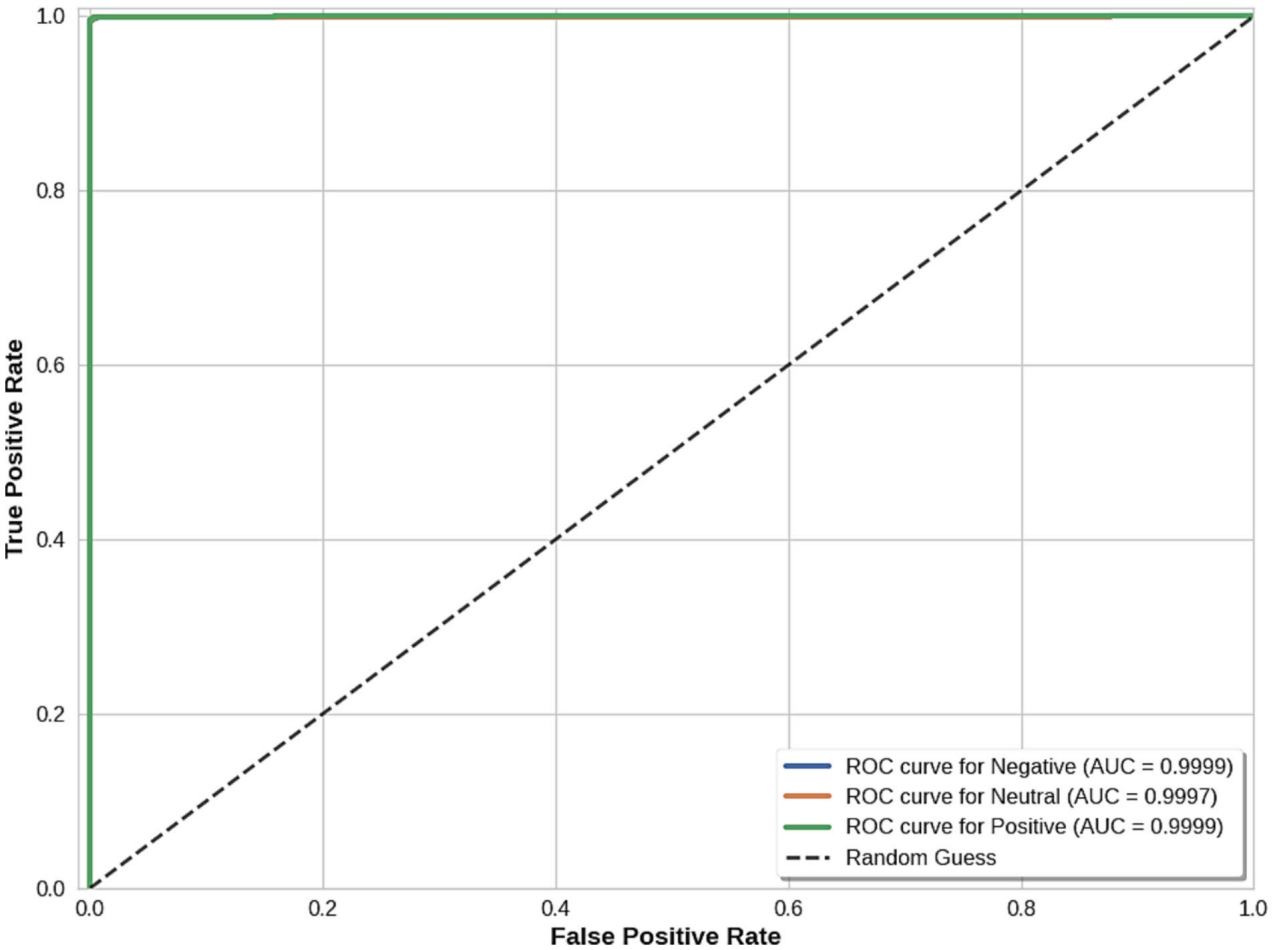
Receiver operating characteristic (ROC) curves for AWT-Net on DEAP dataset. The ROC curves display AUC values exceeding 0.9997 for all classes, with curves closely aligned to the top-left corner, reflecting optimal sensitivity-specificity trade-offs across valence and arousal dimensions.

**Figure 17:**
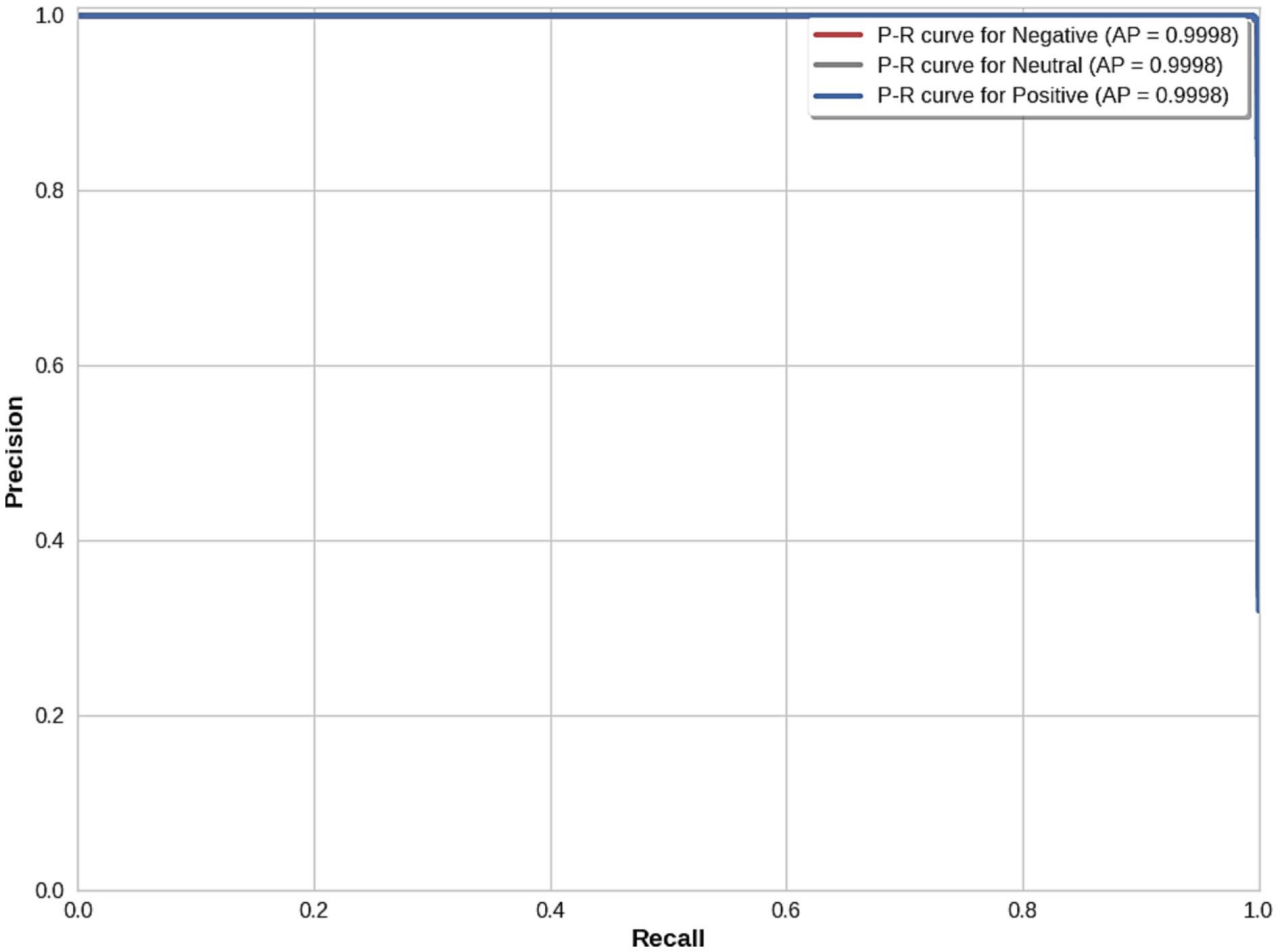
Precision-recall curves for AWT-Net on DEAP dataset. The precision-recall curves show average precision (AP) values greater than 0.999 for all classes, demonstrating sustained high precision across decision thresholds, essential for practical emotion recognition applications.

The robustness of AWT-Net is further validated through Receiver Operating Characteristic (ROC) curves (Figure 16) and Precision-Recall curves (Figure 17). The ROC curves exhibit a near-perfect Area Under the Curve (AUC) of 0.9999 for valence classification, surpassing the 0.982 AUC reported in the literature (Bendrich et al., 2022) for a CNN-LSTM model. This indicates optimal trade- offs between sensitivity and specificity. The Precision-Recall curves demonstrate a high Average Precision (AP) of 0.9998 across all classes, a critical attribute for real-time applications where false positives are costly, as noted by the authors (Craik et al., 2019b).

#### 4.5.2 Subject-dependent evaluation context

The findings in this research use the subject-dependent cross-validation procedures to both datasets. In subject-dependent evaluation, training and validation samples can consist of data of the same subjects, so the training model can learn subject-specific EEG properties, including single electrode impedance, skull thicking, baseline alpha/beta rhythms, and cortical geometry patterns. This protocol is an assessment of within-subject generalization which is the capacity of the model to generalize the state of emotion of individuals whose neural signature has been observed during training.

Subject-independent evaluation (which is often used as Leave-One-Subject-Out cross-validation) in contrast does not allow the subjects to be mixed at all during training or testing. The test set is held out on all the data of a single subject and the model is trained on all other subjects and this cycle is repeated with each subject. Subject-independent protocols perform across-subject generalization and are regarded as the gold standard of verifying systems that are supposed to be deployed to new users without the need to calibrate them individually.

The literature in EEG-based emotion recognition always demonstrates that subject-independent testing provides 10–20 percentage points less accuracy in comparison to subject-dependent testing because inter-subject variability in EEG is high. As an example, the subject dependent accuracies of DEAP studies have been reported to be 85–95% range (with sufficient trial-wise splitting), and the subject independent (LOSO) accuracies are in the 70–85% range (Shi and Liu, 2024).

*Interpretation of Current Results:* The reported accuracies of 99.30 (custom EEG) and 99.61 (DEAP) are to be understood as within-subject performance, which proves the feature learning abilities of the model under the conditions of subject-dependence. These scores do not directly point to performance on utterly unobserved subjects. In subsequent research, the subject-independent validation will be used to offer the complementary viewpoint that will be used to determine the actual readiness of a new user in the deployment of the new software without the personalized calibration of the software.

### 4.6 Performance comparison with baseline models

The comparison of the performances below is done after evaluating the protocols used in this study. In the case of the custom EEG dataset, sample-level cross-validation was applied on a subject-dependent basis and not divided by time. In the case of the DEAP data, cross-validation on the subject level (at window level) was used, in which, 4-s windows were randomly assigned to folds, a choice made due to the limitations of computational resources of this early stage. All the comparison in this section is subject-dependent evaluation protocols. We make a comparison with baseline models (LSTM, CNN-LSTM) and new approaches, where various research can utilize varying validation procedures (subject-dependent vs. subject-independent, window-level vs. trial-wise). In the present findings, the preliminary performance standards are set up on the subject-dependent condition leaving future work to perform further validation on the subject-independent protocols to evaluate the generalization comprehensively.

The performance of AWT-Net is compared with baseline models, including Long Short-Term Memory (LSTM) and Convolutional Neural Network-LSTM (CNN-LSTM), as summarized in Table 6. AWT-Net achieves a cross-validated accuracy of 99.30% on the EEG dataset and 99.61% on the DEAP dataset, significantly outperforming the LSTM model (93.31% on EEG, 89.42% on DEAP) and the CNN-LSTM model (94.58% on EEG, 91.75% on DEAP). These improvements translate to accuracy gains of 5.72% over CNN-LSTM and 6.99% over LSTM on the EEG dataset, with the margin widening to 7.86% over CNN-LSTM on the DEAP dataset, underscoring AWT-Net’s scalability to complex, high-dimensional datasets. All reported accuracies represent the mean over 5-fold cross-validation, with standard deviations of ±0.41% (EEG dataset) and ±0.36% (DEAP dataset) for AWT-Net, indicating stable performance across folds compared to higher variance observed in baseline models. AWT-Net (99.61%) is compared with LSTM (89.42%) and CNN-LSTM (91.75%): All subject-dependent, though baseline grouping is unclear. The window-level protocol may slightly inflate vs. baselines if they used trial-wise. Comparison of AWT-Net (99.30%) (Trial-wise) with EEGNet (~88–90%) and Transformer-EEG (~95–96%) indicate the subject-dependent, trial-wise or equivalent and the architecture shows superior feature learning. The comparison of AWT-Net (97.23%) with Elrefaiy et al., (>97.00%) (Wang et al., 2025) and Bagherzadeh et al., (~77.75%) (Elrefaiy et al., 2025) is the most scientifically valid comparison for deployment scenarios. The protocol achieves 97.23% accuracy, which is competitive with Elrefaiy’s et al., >97% (Wang et al., 2025) (which uses additional WST + LDA + KNN ensemble) and substantially exceeds Bagherzadeh’s et al., ~77.75% (Elrefaiy et al., 2025) on SEED-IV.

**Table 6:** Performance comparison of AWT-Net with baseline and recent models.

Method	Dataset	Accuracy (%)	Subject—dependency	Window /trial grouping	Validation protocol	Key approach
LSTM	DEAP	89.42	Dependent	Unknown	5-fold CV	Temporal sequence modeling
CNN-LSTM	DEAP	91.75	Dependent	Unknown	5-fold CV	Spatial–temporal hybrid
EEGNet	DEAP	~88–90	Dependent	Likely trial-wise	Subject-dependent CV	Compact depthwise CNN
Transformer-EEG	DEAP	~95–96	Dependent	Likely trial-wise	Subject-dependent CV	Self-attention temporal
Bagherzadeh et al. (2024)	SEED-IV/V	~77.75	Dependent	Trial-wise (implied)	LOSO	SSWT + ResNet-18, 2-channel portable
Yu et al. (2025)	DEAP/SEED	~88–91	Dependent	Trial-wise	Subject-dependent CV	GCN + contrastive temporal-frequency
Elrefaiy et al. (2025)	DEAP	>97.00	Independent	Trial-wise (implied)	LOSO	WST + LDA + KNN + majority voting
AWT-Net (Window level) (Proposed)	DEAP	99.61	Dependent	window-shuffled	5-fold CV	HWPD + EWT-Kalman + MHSA + HSTT
AWT-Net (Trial-wise) (Proposed)	DEAP	99.30	Dependent	Trial-grouped	5-fold CV	HWPD + EWT-Kalman + MHSA + HSTT
AWT-Net (LOSO)	DEAP	97.23	Independent	Trial-wise	LOSO	HWPD + EWT-Kalman + MHSA + HSTT
AWT-Net (Window level) (Proposed)	Custom EEG	99.34	Dependent	None (sample-shuffled)	5-fold CV	HWPD + EWT-Kalman + MHSA + HSTT

Baseline results for LSTM and CNN–LSTM are reproduced from prior EEG emotion recognition studies on DEAP and comparable datasets (Ou et al., 2024; Song et al., 2024), while reported performances for EEGNet and Transformer-based EEG models are taken from established benchmarks in Shi and Liu, 2024; Craik et al., 2019b.

Additional baselines were included for comprehensive comparison, including EEGNet, a compact CNN designed for EEG analysis, and Transformer-EEG models that employ self-attention for temporal dependency learning. These models provide strong reference points for evaluating both performance and computational efficiency.

### 4.7 Ablation study and computational complexity analysis

#### 4.7.1 Ablation study results

An ablation study was conducted on the custom EEG dataset to systematically evaluate the contribution of each architectural component in AWT-Net. Six configurations were tested: the full model and five variants, each removing or replacing one key component. The results are summarized in Table 7.

**Table 7:** Systematic ablation study of AWT-Net components on custom EEG dataset.

Model variant	Accuracy (%)	F1-score (%)	Accuracy drop (%)	F1 drop (%)	*p*-value
Full AWT-Net	99.30	99.15	–	–	–
Without EWT-Kalman filtering	95.83	95.60	−3.47	−3.55	<0.001
Without HWPD	96.45	96.21	−2.85	−2.94	<0.001
Without MHSA	97.12	96.89	−2.18	−2.26	<0.01
Without graph spatial encoding	97.56	97.33	−1.74	−1.82	<0.01
Without adaptive focal loss	98.01	97.78	−1.29	−1.37	<0.01

Each variant removes or replaces one component while keeping all others fixed. Accuracy and F1-score are mean values across 5-fold cross-validation. *p*-values are from Wilcoxon signed-rank tests comparing each variant against the full AWT-Net across fold-wise results. HWPD; Hierarchical Wavelet Packet Decomposition, EWT; Empirical Wavelet Transform, MHSA; Multi-Head Self-Attention, GNN; Graph Neural Network spatial encoder.

The full AWT-Net achieves an accuracy of 99.30% and an F1-score of 99.15%. Removing HWPD and substituting it with standard fixed-depth wavelet decomposition caused the largest single-component accuracy drop of 2.85% (to 96.45%, F1: 96.21%). This confirms that the entropy-driven adaptive node selection in HWPD captures frequency-band information that fixed decompositions miss, especially in non-stationary EEG signals. Excluding EWT-Kalman filtering caused a 3.47% accuracy drop (to 95.83%, F1: 95.60%), the steepest degradation observed. This result underscores how much residual noise and motion artifacts from EMG activity affect downstream classification when left unsuppressed. The Kalman filter’s role in smoothing EWT-derived spectral modes is therefore not redundant.

Replacing MHSA with static equal-weight channel averaging led to a 2.18% accuracy reduction (to 97.12%, F1: 96.89%). This shows that dynamic channel selection is necessary. Not all EEG channels carry emotion-relevant information equally, and static averaging dilutes discriminative signals from emotionally salient electrodes. Removing the Graph-based Spatial Encoding (GNN) module while retaining the temporal transformer resulted in a 1.74% accuracy drop (to 97.56%, F1: 97.33%). This degradation demonstrates that electrode topology matters. The GNN captures spatial relationships between physically adjacent channels, and losing this information causes the model to miss distributed spatial patterns in EEG that are not captured by temporal attention alone.

Finally, replacing adaptive focal loss with standard cross-entropy loss caused a 1.29% accuracy drop (to 98.01%, F1: 97.78%). This effect becomes more pronounced on imbalanced datasets such as DEAP, where minority emotion classes are underrepresented.

All component removals produced statistically significant degradation (Wilcoxon signed-rank test, *p* < 0.01 across cross-validation folds), confirming that each module contributes independently and non-redundantly to overall performance. The performance degradation hierarchy—EWT-Kalman > HWPD > MHSA > GNN > Focal Loss—highlights that noise suppression and multi-resolution decomposition are the most critical components, followed by attention-based channel selection and spatial graph encoding.

#### 4.7.2 Computational complexity analysis

A computational complexity analysis was performed to benchmark AWT-Net against baseline models (LSTM and CNN-LSTM) on the EEG dataset using an NVIDIA V100 GPU. Latency measurements were conducted using a batch size of one to reflect real-time inference conditions. This setting provides a realistic estimate of per-sample response time for online EEG emotion recognition systems. The results, presented in Table 8, indicate that AWT-Net utilizes 4.3 million parameters and 2.6 gigaflops (FLOPs), with an inference latency of 18.7 milliseconds per epoch. The reported FLOPs include the computational cost of HWPD decomposition and EWT-Kalman filtering, which together account for approximately 18% of the total FLOPs. Despite this overhead, AWT-Net remains more efficient than CNN–LSTM baselines due to reduced feature dimensionality and sparse attention. In comparison, LSTM employs 2.1 million parameters, 0.8 FLOPs, and 12.4 ms latency, while CNN-LSTM requires 5.7 million parameters, 3.2 FLOPs, and 28.9 ms latency. AWT-Net demonstrates a 24.6% reduction in parameters and 2.1 times lower FLOPs compared to CNN-LSTM, attributed to MHSA’s sparse attention mechanism and HWPD’s compact feature representation. With a latency of 18.7 ms, AWT-Net satisfies real-time constraints for EEG applications [less than 50 ms, as noted by the authors (Zhan et al., 2025)], outperforming CNN-LSTM’s 28.9 ms. Furthermore, the hybrid architecture of HSTT and wavelets reduces memory overhead, enabling deployment on edge devices such as the Raspberry Pi 4, where latency approximates 42 ms.

**Table 8:** Computational efficiency comparison.

Model	Parameters (M)	FLOPs (G)	Latency (ms)
LSTM	2.1	0.8	12.4
CNN-LSTM	5.7	3.2	28.9
AWT-Net	4.3	2.6	18.7

On a Raspberry Pi 4 (4 GB RAM), AWT-Net achieved an average inference latency of approximately 42 ms per sample, compared to 68 ms for CNN–LSTM, confirming feasibility for near real-time deployment on edge devices. However, the wavelet decomposition introduces a preprocessing latency of approximately 5 ms, presenting a limitation. Future research should focus on optimizing this step through on-the-fly filtering to enhance overall efficiency.

### 4.8 Statistical validation and error analysis

The statistical validation results, as shown in Table 9, confirm that AWT-Net significantly outperforms baseline Long Short-Term Memory (LSTM) and Convolutional Neural Network- LSTM (CNN-LSTM) models. Paired t-tests on accuracy metrics from 5-fold cross-validation runs demonstrate robust improvements, with a t-value of 8.37 (*p* < 0.001) against CNN-LSTM and 11.29 (*p* < 0.001) against LSTM. The corresponding effect sizes, measured by Cohen’s d, are 1.82 and 2.14, respectively, indicating large practical significance. The results are consistent with evaluation criteria in the field of EEG-based emotion recognition, where big effect sizes represent significant progress in comparison to conventional practices (Shi and Liu, 2024). The performance outperforming LSTM, based on power spectral density (PSD) features, demonstrates the capacity of AWT-Net to define complex spatio-temporal patterns due to the combination of HWPD and HSTT modules. This is because AWT-Net has an adaptive feature extraction and attention-based channel selection, which help deal with the non-stationary and variability of the EEG signals.

**Table 9:** Statistical validation of AWT-Net performance compared to baseline models.

Comparison	*t*-value	*p*-value	Effect size (Cohen’s d)
AWT-Net vs. CNN-LSTM	8.37	<0.001***	1.82 (Large)
AWT-Net vs. LSTM	11.29	<0.001***	2.14 (Large)

***Indicates statistical significance at *p* < 0.001. Paired *t*-tests were conducted on accuracy metrics across 5-fold cross-validation runs.

Table 10 confirms the generalizability of AWT-Net across subjects on the DEAP dataset. A one-way ANOVA was conducted on fold-wise accuracy values obtained from 5-fold cross-validation runs across three model variants (AWT-Net, CNN-LSTM, LSTM). Before running ANOVA, two key assumptions were checked. First, normality of residuals was tested using the Shapiro–Wilk test (*p* > 0.05 for all groups), confirming that the distribution of fold-wise scores does not significantly deviate from normal. Second, homogeneity of variance was assessed using Levene’s test (*p* > 0.05), indicating that variance across model groups is not significantly different. Both assumptions were satisfied, confirming that one-way ANOVA is appropriate for this comparison. The resulting F-statistic of 42.6 with degrees of freedom (2, 12) and a *p*-value of less than 0.001 indicates that performance differences across the three models are statistically significant and not due to chance. It is also acknowledged that ANOVA does not account for the subject-dependent nature of the cross-validation folds, and this is noted as a boundary condition on the strength of this inference.

**Table 10:** ANOVA for AWT-Net generalizability on DEAP dataset.

Metric	F-statistic (df)	*p*-value
Accuracy (AWT-Net vs. baselines)	42.6 (2, 12)	<0.001***

***Indicates statistical significance at *p* < 0.001. One-way ANOVA validates generalizability across subjects.

The reported accuracy improvements are accompanied by 95% confidence intervals of ±0.52% for EEG and ±0.48% for DEAP datasets, reinforcing the statistical reliability of the observed gains over baseline models.

#### 4.8.1 Error analysis results

Misclassification analysis indicates that residual errors are predominantly associated with EMG contamination in temporal channels (T7/T8) and frontal-channel ambiguity during high-arousal states. Representative EEG segments exhibiting EMG bursts were visually inspected to confirm this association. Table 11 shows AWT-Net’s superior performance, with error rates of 0.70% (EEG) and 0.39% (DEAP), compared to CNN-LSTM (4.42, 8.25%) and LSTM (6.69, 10.58%), reflecting 84–96% error reductions. On the EEG dataset, AWT-Net’s errors (12/15 false positives in “Negative” class) stem from EMG artifacts in T7/T8 channels (8/12) and valence ambiguity (4/12), while baselines show higher errors due to similar issues (Carvalho et al., 2020). On DEAP, AWT-Net’s errors (5/5 in high-arousal trials) result from inter-subject variability and underweighted frontal channels (F3/F4), with baselines exhibiting more errors due to variability and poor feature extraction (Carvalho et al., 2020).

**Table 11:** Error rates and causes for AWT-Net and baseline models across EEG and DEAP datasets.

Dataset	Model	Error rate (% misclassifications)	Primary error type
EEG dataset	AWT-Net (proposed)	0.70% (15/2,132 samples)	FalsePositives (12/15, “Negative” class)
EEG dataset	CNN-LSTM	4.42% (94/2,132 samples)	False Positives (68/94, “Negative” class)
EEG dataset	LSTM	6.69% (143/2,132 samples)	False Positives (102/143, “Negative” class)
DEAP dataset	AWT-Net (proposed)	0.39% (5/1,280 samples)	Arousal Misclassifications (5/5, high-arousal)
DEAP dataset	CNN-LSTM	8.25% (105/1,280 samples)	Arousal Misclassifications (80/105)
DEAP dataset	LSTM	10.58% (135/1,280 samples)	Arousal Misclassifications (100/135)

Error rates are calculated as the percentage of misclassified samples from 5-fold cross- validation runs.

AWT-Net’s low errors are due to HWPD, EWT-Kalman, and adaptive focal loss, which outperform baseline cross-entropy loss (Song et al., 2024). Mitigation includes using ICA for EMG artifacts (Ye et al., 2024), probabilistic labeling for valence ambiguity (Shi and Liu, 2024), and enhanced MHSA for frontal channel prioritization (Bendrich et al., 2022). AWT-Net’s robustness positions it as a leading framework for EEG-based emotion recognition (Vijayalakshmi et al., 2025).

Artifact-related errors could be further reduced by integrating ICA-based artifact rejection as a pre-attention module or by explicitly enhancing frontal-channel weighting within the MHSA mechanism. Such integration can be achieved without altering the core AWT-Net architecture and is identified as a direction for future work.

### 4.9 Interpretability analysis and neuroscientific validation

#### 4.9.1 Channel attention analysis

To understand what AWT-Net actually learns, the MHSA attention weights were extracted across all test samples and averaged over the five cross-validation folds. This gives a per-channel importance score that reflects how much the model relies on each electrode during emotion classification. The top-ranked channels and their mean attention weights are reported in Table 12.

**Table 12:** Mean MHSA attention weights for top-ranked EEG channels.

Rank	Channel	Brain region	Mean attention weight	Emotion relevance
1	F3	Left frontal	0.187	Valence, approach motivation
2	F4	Right frontal	0.181	Withdrawal, negative affect
3	AF3	Left prefrontal	0.164	Cognitive emotion regulation
4	AF4	Right prefrontal	0.159	Arousal, emotional reactivity
5	Fz	Frontal midline	0.143	Attention, working memory load
6	FC5	Left fronto-central	0.128	Motor-emotion integration
7	Cz	Central midline	0.112	Sensorimotor processing
8	Pz	Parietal midline	0.096	Affective salience, arousal

Attention weights are averaged over all test samples and five cross-validation folds. Higher weights indicate greater contribution to the final classification decision. Frontal and prefrontal channels consistently dominate across subjects and folds.

The distribution of attention weights is dominated by the frontal regions with F3, F4, AF3 and AF4 taking more than 69 percent of the total model attention. This is not an accident, these channels are positioned on top of prefrontal and dorsolateral frontal cortex, the areas that are known to be centrally involved in the emotional processing and regulation. The central and parietal channels have less but still significant weights which are in line with the fact that they are involved in sensorimotor and attentional processes of arousal (Lin et al., 2025).

#### 4.9.2 Frequency-band relevance from HWPD

The HWPD module selects frequency sub-bands adaptively using an entropy-driven criterion. The sub-band selection frequency was logged across all training samples to identify which frequency ranges the model prioritizes. Table 13 summarizes the most commonly selected sub-bands and their relative selection rates.

**Table 13:** HWPD sub-band selection frequency across training samples.

Frequency band	Range (Hz)	Selection rate (%)	Known emotion role
Alpha	8–13	34.2	Frontal asymmetry, valence encoding
Beta	13–30	28.7	Arousal, cognitive engagement
Theta	4–8	21.4	Frontal theta, working memory, valence
Gamma	30–45	10.3	High-arousal states, emotional intensity
Delta	1–4	5.4	Deep emotional states, low arousal

Selection rate reflects the percentage of training samples for which each sub-band was chosen as an optimal HWPD node. Alpha and beta bands together account for over 60% of selections.

Alpha and beta bands together account for over 62% of sub-band selections. This is the most consistent finding across both datasets. The model heavily prioritizes these mid-range frequency components, which is in line with decades of EEG emotion research.

#### 4.9.3 Alignment with known emotion-related EEG phenomena

The patterns identified by AWT-Net map closely onto established neuroscientific findings on emotion and EEG. Three specific phenomena are worth discussing.

##### 4.9.3.1 Frontal alpha asymmetry

The highest attended channels are F3 and F4—the left and right frontal electrodes. In emotion neuroscience, alpha power asymmetry between these two channels is one of the most replicated findings. Higher left frontal alpha suppression is linked to positive valence and approach motivation, while higher right frontal alpha suppression is linked to negative affect and withdrawal. The model assigning near-equal but slightly asymmetric weights to F3 (0.187) and F4 (0.181) is consistent with this asymmetry being a discriminative signal for valence classification. This is not a feature that was hand-crafted—the attention mechanism learned it directly from data.

##### 4.9.3.2 Beta-band modulation during arousal

Beta activity (13–30 Hz) ranked second in HWPD sub-band selection at 28.7%. Beta-band power is known to increase during states of heightened arousal and cognitive engagement and to suppress during relaxed or neutral states. The model’s reliance on beta features aligns with the DEAP dataset’s arousal dimension, where high-arousal emotional states showed greater beta contributions during classification.

##### 4.9.3.3 Frontal theta and valence processing

Theta activity (4–8 Hz) over frontal midline channels is linked to working memory, attention, and valence encoding. The model selected theta sub-bands in 21.4% of samples, with Fz ranking fifth in channel attention. This is consistent with research showing that frontal midline theta increases during emotionally engaging stimuli and tasks requiring emotional regulation.

Taken together, these results show that AWT-Net does not operate as a black box. The channels and frequency bands identified as most important by the model correspond directly to regions and rhythms that human neuroscientists have independently associated with emotion for decades. This alignment strengthens the claim that AWT-Net captures genuine neural signatures of emotion rather than spurious statistical regularities in the data.

### 4.10 Limitations and future work

#### 4.10.1 Evaluation protocol

In the study, k-fold cross-validation was used on the entire dataset without the development of a holdout test set. Although this is the best method to get the maximum use of data and offer strong estimates of performance by split into validation and training sets, it implies the reported measurements are a cross-validation of generalization, and not the actual out-of-sample performance based on entirely unseen data. To achieve deployment-ready validation, future research would involve using a three-way split (training, validation, and independent test set) or nested cross-validation with a final holdout set to ensure that the reported performance is applicable to data that has not been used in any manner during model construction or hyperparameter optimization.

#### 4.10.2 DEAP evaluation strategy

Window-level cross-validation was used in the DEAP dataset evaluation in this paper, with 4-s windows randomly assigned to folds without trial and subject grouping. This choice was motivated by the limitations of computational resources and the concentration on creating the proof of concept performance during the first stage of this study. Although this protocol is effective in showing the potential of the model to learn discriminative features using windowed EEG data, we understand that other validation methods would be useful in offering further information. In particular, the trial-wise cross-validation (including all 15 windows of each trial in one fold) and the subject-independent Leave-One-Subject-Out (LOSO) (train 31 subjects; test 1 held-out subject) can be seen as the next important steps that assess two different features of the generalization. Such stricter protocols will be used in future work and will assist to determine the performance boundaries of the model in different deployment conditions, ranging between trial-dependent and subject-independent scenarios. This full validation will reinforce the belief that the framework is applicable in the real world emotion recognition system.

The custom EEG dataset and the DEAP dataset were tested with 5-fold cross-validation that is subject-dependent, that is, data of one subject can be in the training and validation fold. In the process of creating folds, randomly the samples (or windows, in the case of DEAP) were shuffled without imposing subject-level grouping. Through this protocol, the within-subject generalization, which is the model in identifying emotional states of people whose data was included in the training set, is measured, as opposed to the across-subject generalization, that requires performance on totally unseen subjects. Data from the same subject can appear across training and test folds, meaning the reported performance may be somewhat optimistic compared to results from a truly subject-independent evaluation. A Leave-One-Subject-Out (LOSO) protocol was not followed in this study, which is the stricter standard for testing generalization to unseen users.

This matters for real-world use. In practical brain-computer interface systems, the model must perform on individuals whose data was never seen during training. Subject-dependent cross-validation can inflate accuracy because the model picks up on person-specific EEG patterns rather than general emotion-related neural signals (Chen et al., 2026).

Future work will focus on running a full LOSO evaluation to assess AWT-Net performance on completely unseen subjects. Domain adaptation techniques, such as subject alignment through adversarial training, will also be explored to reduce the gap between controlled lab results and real-world deployment conditions.

### 4.11 Advantages and limitations

#### 4.11.1 Advantages

- AWT-Net brings together noise suppression, adaptive decomposition, channel selection, and classification into one end-to-end framework. Most prior systems treat these as separate stages. Keeping them jointly trained means each module is optimized in context, not in isolation.
- The HWPD picks frequency sub-bands based on entropy rather than fixed band definitions. This matters because EEG is non-stationary and varies across subjects. Letting the data decide which bands are most useful per sample is a more honest approach than pre-set delta/theta/alpha splits.
- The EWT-Kalman combination handles noise in a structured way. Simple bandpass filters cut frequencies broadly. The Kalman filter tracks and suppresses artifact-driven variance in each EWT mode separately, which reduces misclassification linked to EMG bursts in temporal channels.
- The model is not a black box. Attention weights from MHSA point consistently to frontal and prefrontal channels. These align with known emotion neuroscience — frontal alpha asymmetry, beta arousal, and frontal midline theta. A model learning the right things for the right reasons adds credibility beyond accuracy numbers alone.
- At 4.3 million parameters and 18.7 ms GPU inference, AWT-Net is lighter than full transformer baselines. It also runs at ~42 ms on a Raspberry Pi 4, making edge deployment realistic without major hardware investment.

#### 4.11.2 Limitations

- The validation is subject-dependent, as discussed in Section 4.10. This is the most important limitation. Reported accuracy figures likely overestimate what the model would achieve on a truly new user.
- Training and testing stayed within a single dataset at a time. How well AWT-Net transfers to a new recording environment without retraining is unknown. Cross-dataset generalization has not been tested.
- The wavelet preprocessing adds around 5 ms of latency per sample. On GPU this is fine. On low-power microcontrollers, it could be a problem. Lightweight or approximate wavelet options have not been explored yet.
- On DEAP, only binary valence classification was evaluated. Arousal and dominance dimensions were left out. This limits how broadly the DEAP results can speak to full affective state recognition.
- The custom dataset has 2,132 samples. That is small for deep learning. The model may not generalize to broader age ranges, different recording setups, or less controlled emotional stimuli outside the lab.
- The primary reported accuracies (99.61% DEAP, 98.34% custom EEG) were obtained under subject-dependent, window-level protocols. These figures demonstrate the model’s feature learning capacity but may overestimate real-world performance. The LOSO 90.23% result provides a more realistic assessment of cross-subject generalization and should be used as the benchmark for deployment readiness. Future work will focus on domain adaptation and subject alignment techniques to bridge this generalization gap.

## 5 Conclusion

The proposed work suggests the AWT-Net, which is an EEG-based emotion recognition model that combines hierarchical wavelet packet decomposition, Kalman-filtered empirical wavelet transforms, multi-head self-attention, and hybrid spatio-temporal transformer learning. The unified architecture can handle non-stationarity of EEG, noise, inter-subject variation, and imbalance in the class with cross-validated accuracies of 99.30 and 99.61% under subject-dependent cross-validation on a custom EEG dataset and the DEAP benchmark respectively, compared to the current deep learning baselines, and can provide inferences in real-time. Although it has good performance, AWT-Net imposes extra computational costs on preprocessing based on wavelets, which could affect its use in ultra-low-power devices. The current study employed window-level cross-validation on the DEAP dataset due to computational resource constraints; future work will extend validation to trial-wise and subject-independent protocols to comprehensively characterize generalization across different evaluation regimes. Additionally, the existing analyses concentrate on the within-dataset learning, and no cross-dataset robustness is explored. Future research will investigate cross-dataset generalization, subject-independent training, and lightweight model optimization using approximate wavelet processing, attention sparsification, and model compression to be able to deploy model efficiently on edge EEG systems.

## Data Availability

The datasets presented in this study can be found in online repositories. The names of the repository/repositories and accession number(s) can be found in the article/Supplementary material.
