# Supplementary material for: Modeling multiscale neural dynamics for EEG-based emotion recognition using an attentive wavelet–transformer framework
Source: Front Comput Neurosci. 2026 Jun 9;20:1775449. doi: 10.3389/fncom.2026.1775449 (PMC13286962; doi:10.3389/fncom.2026.1775449)
Supplement: Supplementary file 1 [file Supplementary_file_1.docx]

**Appendix A: Dataset and Implementation Details**

Due to confidentiality, the full custom dataset cannot be publicly released. A subset of anonymized sample images is available at [https://github.com/rssoundariya19/EEG-Emotion- recognition]. The implementation code for AWT-Net, is publicly accessible at [https://github.com/rssoundariya19/EEG-Emotion-recognition], enabling replication with alternative datasets.

**Appendix B: Reproducibility and Implementation Details**

This section consolidates all implementation details needed to reproduce the reported experiments. All experiments were run using the same configuration across both datasets unless stated otherwise.

**B.1 Hardware Environment**

**Table B1. Hardware and Software Environment**

| **Component** | **Specification** |
| --- | --- |
| GPU | NVIDIA Tesla V100 (32 GB HBM2) |
| CPU | Intel Xeon Gold 6148 (20 cores, 2.4 GHz) |
| RAM | 128 GB DDR4 |
| Storage | 2 TB NVMe SSD |
| Operating System | Ubuntu 20.04 LTS |
| Deep Learning Framework | PyTorch 1.13.1 |
| Python Version | Python 3.9.7 |
| CUDA Version | CUDA 11.7 |
| Additional Libraries | NumPy 1.23, SciPy 1.9, PyWavelets 1.4, Scikit-learn 1.1 |

*Note: Edge deployment tests were additionally run on a Raspberry Pi 4 (4 GB RAM, ARM Cortex-A72, 64-bit OS) to assess feasibility for embedded applications.*

**B.2 Hyperparameter Settings**

All hyperparameters were fixed before training and not tuned between folds. The values below apply to both the custom EEG dataset and the DEAP dataset unless a dataset-specific value is noted.

**Table B2. Hyperparameter Configuration for AWT-Net**

| **Hyperparameter** | **Value** | **Notes** |
| --- | --- | --- |
| Optimizer | Adam | Default betas: β1=0.9, β2=0.999 |
| Learning Rate | 0.001 | Fixed; no scheduler used |
| Batch Size | 32 | Single sample (batch=1) used for latency tests |
| Number of Epochs | 100 (EEG), 15 (DEAP) | DEAP converged faster due to simpler binary task |
| Cross-Validation Folds | 5 | Stratified split by class label |
| HWPD Decomposition Depth | 4 levels | Entropy-driven node selection at each level |
| HWPD Wavelet Basis | Daubechies-4 (db4) | Selected for its compact support and smoothness |
| EWT Spectral Modes | Adaptive (3–7 modes) | Determined per sample from Fourier spectrum |
| Kalman Process Noise (Q) | 1e-4 | Initialized from empirical EEG variance |
| Kalman Observation Noise (R) | 1e-2 | Fixed across all channels |
| MHSA Attention Heads | 8 | Query/key dimension dk = 64 per head |
| Transformer Encoder Layers | 3 | Each with residual connection and layer norm |
| GNN Hidden Dimension | 128 | Learned adjacency matrix; ReLU activation |
| Dropout Rate | 0.3 | Applied after each transformer block |
| Focal Loss Gamma (γ) | 2.0 | Consistent with empirical EEG imbalance findings |
| Focal Loss Class Weights | Inverse class frequency | Recomputed per fold |
| Weight Initialization | Xavier uniform | Applied to all linear layers |
| Gradient Clipping | Max norm = 1.0 | Prevents gradient explosion in transformer layers |
| Window duration | 4 seconds | DEAP only |
| Window size | 512 timesteps (at 128 Hz) | DEAP only |
| Window overlap | 0% (non-overlapping) | DEAP only |
| Windows per trial | 15 | DEAP only |
| Total windows | 19,200 (from 1,280 trials) | DEAP only |
| Fold assignment | Window-level random shuffle | DEAP only |

*Note: Due to computational resource constraints, cross-validation folds were created by randomly shuffling the 19,200 windows without trial-wise grouping. This window-level evaluation protocol enabled efficient training and provided initial performance benchmarks. Future studies will implement trial-wise cross-validation (grouping windows from the same trial) and subject-independent LOSO evaluation to comprehensively assess generalization capabilities.*

**B.3 Training Time**

**Table B3. Training and Inference Time for AWT-Net**

| **Metric** | **Custom EEG Dataset** | **DEAP Dataset** |
| --- | --- | --- |
| Total Training Time (GPU) | ~47 minutes | ~31 minutes |
| Training Time per Epoch (GPU) | ~28 seconds | ~124 seconds |
| Total Training Time (5-fold CV) | ~3.9 hours | ~2.6 hours |
| Inference Latency per Sample (GPU) | 18.7 ms | 18.7 ms |
| Inference Latency per Sample (Raspberry Pi 4) | ~42 ms | ~42 ms |
| Preprocessing Time per Sample (Wavelet) | ~5 ms | ~5 ms |

*Note: Training time per epoch is higher for DEAP due to larger input dimensionality (40 channels × 8,064 timesteps versus the custom dataset's lower temporal resolution). All times are averaged over five cross-validation folds and measured on the NVIDIA V100 GPU unless otherwise stated.*

**Table B4: Cross-Validation Protocol Specifications**

| **Aspect** | **Custom EEG Dataset** | **DEAP Dataset** |
| --- | --- | --- |
| Protocol Type | Subject-dependent | Subject-dependent |
| Cross-validation | 5-fold | 5-fold |
| Fold assignment unit | Sample (no segmentation) | Window (4-sec segments) |
| Subject grouping | None (random shuffle across subjects) | None (random shuffle across subjects) |
| Subjects in dataset | 28 | 32 |
| Can same subject appear in train & validation? | Yes | Yes |
| Can same trial appear in train & validation? | N/A (no trials) | Yes (windows from same trial can split) |
| Generalization measured | Within-subject | Within-subject |

*Note: Both evaluations use subject-dependent protocols. Subject-independent (LOSO) validation, where all data from one subject is held out completely, is planned for future work and would provide stricter assessment of cross-subject generalization.*

**B.4 Reproducibility Notes**

*Cross-validation implementation details:* For both datasets, standard k-fold cross-validation (k=5) was implemented using scikit-learn's KFold class with shuffle=True and random_state=42. This ensured that: (1) the dataset was randomly shuffled before splitting, (2) fold assignments were reproducible across runs, and (3) each sample appeared in exactly one validation fold. For the custom EEG dataset, StratifiedKFold was used instead to maintain proportional class distribution (Negative, Neutral, Positive) within each fold. The shuffling step was critical to avoid bias from temporal ordering or subject-specific clustering in the original data files. Performance metrics (accuracy, precision, recall, F1-score) for each fold were computed independently, and the reported mean and standard deviation values represent aggregation across all five validation folds. No preliminary train-test split was performed before cross-validation. The entire dataset (100% of samples) was used for k-fold cross-validation, meaning each sample appeared in exactly one validation fold across the five iterations. This differs from a protocol where the dataset would first be split into 80% train and 20% test, followed by cross-validation only within the 80% training portion. Our reported metrics therefore reflect k-fold cross-validated performance on the full dataset without a separate independent test set. The implementation code, including data loading, model definition, training loop, and evaluation scripts, is available at the public GitHub repository listed in Appendix A. Any researcher can run the full pipeline by cloning the repository and following the README instructions. The custom EEG dataset subset and full DEAP preprocessing scripts are also included to ensure end-to-end replication.
